# Hepatocyte-specific deletion of lysosomal acid lipase leads to cholesteryl ester but not triglyceride or retinyl ester accumulation

**DOI:** 10.1074/jbc.RA118.007201

**Published:** 2019-04-25

**Authors:** Laura Pajed, Carina Wagner, Ulrike Taschler, Renate Schreiber, Stephanie Kolleritsch, Nermeen Fawzy, Isabella Pototschnig, Gabriele Schoiswohl, Lisa-Maria Pusch, Beatrix I. Wieser, Paul Vesely, Gerald Hoefler, Thomas O. Eichmann, Robert Zimmermann, Achim Lass

**Affiliations:** From the ‡ Institute of Molecular Biosciences, NAWI Graz, University of Graz, Heinrichstrasse 31/II,; the § Diagnostic and Research Center for Molecular BioMedicine, Institute of Pathology, Medical University of Graz,; the ‖ Center for Explorative Lipidomics, BioTechMed-Graz, and; ¶ BioTechMed-Graz, 8010 Graz, Austria

**Keywords:** lipid metabolism, liver, lysosome, vitamin A, cholesterol, triglyceride, hepatocytes, hyperlipidemia, metabolic disorder, lysosomal acid lipase, neutral lipid ester metabolism

## Abstract

Lysosomal acid lipase (LAL) hydrolyzes cholesteryl ester (CE) and retinyl ester (RE) and triglyceride (TG). Mice globally lacking LAL accumulate CE most prominently in the liver. The severity of the CE accumulation phenotype progresses with age and is accompanied by hepatomegaly and hepatic cholesterol crystal deposition. In contrast, hepatic TG accumulation is much less pronounced in these mice, and hepatic RE levels are even decreased. To dissect the functional role of LAL for neutral lipid ester mobilization in the liver, we generated mice specifically lacking LAL in hepatocytes (hep-LAL-ko). On a standard chow diet, hep-LAL-ko mice exhibited increased hepatic CE accumulation but unaltered TG and RE levels. Feeding the hep-LAL-ko mice a vitamin A excess/high-fat diet (VitA/HFD) further increased hepatic cholesterol levels, but hepatic TG and RE levels in these mice were lower than in control mice. Performing *in vitro* activity assays with lysosome-enriched fractions from livers of mice globally lacking LAL, we detected residual acid hydrolytic activities against TG and RE. Interestingly, this non-LAL acid TG hydrolytic activity was elevated in lysosome-enriched fractions from livers of hep-LAL-ko mice upon VitA/HFD feeding. In conclusion, the neutral lipid ester phenotype in livers from hep-LAL-ko mice indicates that LAL is limiting for CE turnover, but not for TG and RE turnovers. Furthermore, *in vitro* hydrolase activity assays revealed the existence of non-LAL acid hydrolytic activities for TG and RE. The corresponding acid lipase(s) catalyzing these reactions remains to be identified.

## Introduction

In humans, mutations in the *LIPA* gene (encoding lysosomal acid lipase (LAL))^2^ are causative for hepatic cholesteryl ester (CE) and triglyceride (TG) accumulation, associated with plasma hyperlipidemia (*e.g.* elevated total cholesterol and TG levels), hepatomegaly, and the development of liver disease (*e.g.* elevated serum transaminases, jaundice, steatosis, fibrosis, and cirrhosis) (1, 2). If residual LAL activity of 1–12% is present in affected individuals, the symptoms are less severe and patients are typically diagnosed during early childhood with CE storage disease (CESD) (3, 4). If residual activity is below 1%, the clinical symptoms are very pronounced, and affected individuals do not survive beyond the age of 1 year (1). This severe form of LAL deficiency is termed Wolman disease (5).

In mice and similar to humans, genetic disruption of the *Lipa* gene (LAL-knockout; LAL-ko) causes CE and TG accumulation in the liver, and animals develop hepatomegaly (6). In contrast to humans, however, the phenotype of LAL-ko mice resembles more the clinical symptoms of CESD patients, and mice live up to 7–8 months of age (7). LAL-ko mice show increased hepatic expression of genes involved in fatty acid (FA) and cholesterol (CHOL) biosynthesis (8). These changes in hepatic lipid homeostasis are likely a compensatory mechanism for the lysosomal lipid/CHOL entrapment, further aggravating the lipid accumulation phenotype with age (8). Irrespective of the age and disease progression of the animals, the hepatic CE content is always abundantly increased (*e.g.* 14.7- and 42.5-fold in 1.5- and 8-month-old mice, respectively (7)). Remarkably and in contrast to the pronounced CE accumulation phenotype, younger LAL-ko mice show little (7) or no hepatic TG accumulation (9), while at a more advanced age (8 months) hepatic TG content is increased severalfold (*e.g.* 9.7-fold (7)). Furthermore, it is puzzling that LAL-ko mice exhibit decreased hepatic RE content, although recombinant LAL has been shown to hydrolyze retinyl palmitate (RP), and liver homogenates of LAL-ko mice show 90% lower acid RE hydrolase activity (9, 10). These age-dependent and diverging changes in hepatic neutral lipid ester content (*i.e.* of CE *versus* TG and RE) of LAL-ko mice raise the question whether LAL is limiting for CE hydrolysis and changes in hepatic TG and RE contents are consequences of deregulated CHOL homeostasis. The answer can hardly be deduced from the LAL-ko mouse model because of its severe phenotype, which progresses with age and involves many tissues and organs, inducing a very complex pathology. To limit defective LAL to the liver, we generated mice lacking LAL specifically in hepatocytes (hep-LAL-ko). These mice were expected to exhibit a rather mild hepatic neutral lipid phenotype. Furthermore, the phenotype should be alleviated by the uptake of circulating LAL, derived from other liver cell types and other tissues, similar to that observed in LAL-ko mice with tissue-specific transgenic expression of LAL (11). As expected, we observed a moderate CE accumulation in liver of hep-LAL-ko mice, which aggravated upon vitamin A excess/high-fat diet (VitA/HFD) feeding. Interestingly, under standard chow diet, hepatic TG and RE contents of these mice remained unaltered. Paradoxically, however, liver of hep-LAL-ko mice fed a VitA/HFD exhibited lower hepatic TG and RE contents as compared with littermates. Furthermore, lysosome-enriched fractions of liver from hep-LAL-ko mice on VitA/HFD contained increased acid TG hydrolase activities, which were not inhibited by the presence of the LAL-specific inhibitor Lalistat 2, suggesting the presence of so-far uncharacterized acid lipase(s).

## Results

### Generation of mice lacking LAL specifically in hepatocytes

**Figure 1.**
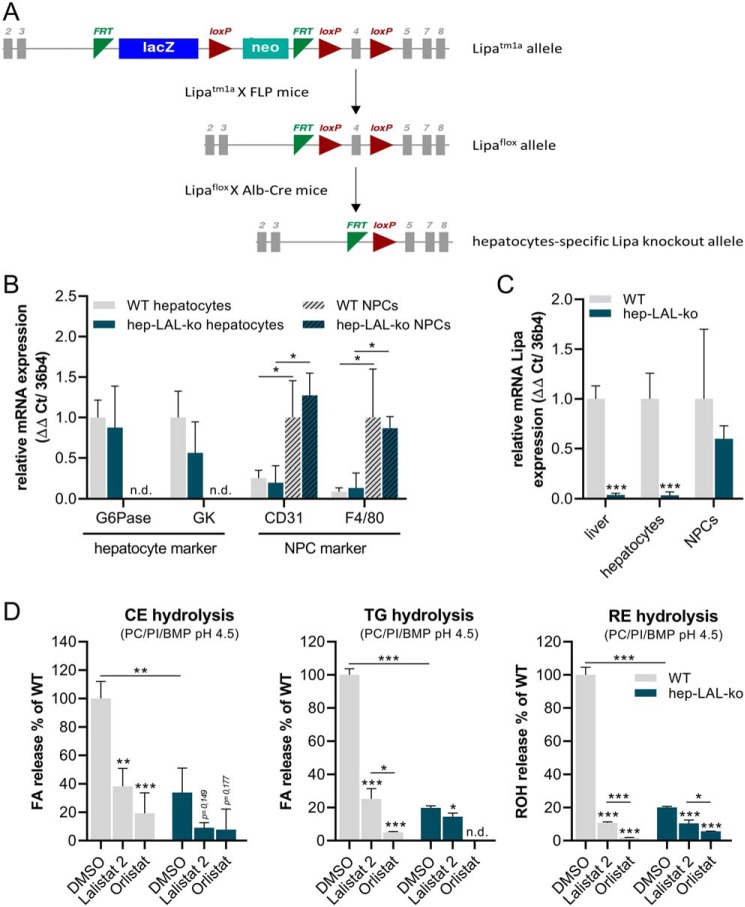
**Breeding strategy and validation of hepatocyte-specific LAL knockout mice.** *A,* Lipa^tm1a^ mice were crossed with FLP-expressing mice. Offspring carrying the floxed Lipa allele (= WT) were crossed with mice expressing Cre recombinase under the control of the albumin promoter (Alb-Cre). Offspring positive for the floxed *Lipa* allele and Alb-Cre lack LAL expression in hepatocytes (hep-LAL-ko). *B–D*, hepatocytes and NPCs were isolated by collagenase digestion. NPCs were cultivated for 5 days. mRNA was transcribed, and expression of marker genes for hepatocytes glucose-6-phosphatase (*G6Pase*) and glycerol kinase (*GK*), for endothelial cells cluster of differentiation 31 (*CD31*), Kupffer cells EGF-like module-containing mucin-like hormone receptor-like 1 (*F4/80*) (*B*), and *Lipa* were determined by qPCR (*C*). Expression levels were calculated by the ΔΔ*Ct* method using *36b4* as ribosomal housekeeping gene. *Lipa* expression was normalized to WT expression levels. *D,* lysates of hepatocytes were incubated with either [^14^C]cholesteryl oleate (*left panel*), [^3^H]triolein *(middle panel*), or retinyl palmitate (*right panel*), respectively, emulsified with PC, PI, and BMP in sodium acetate buffer (pH 4.5), including FA-free BSA. Furthermore, DMSO as control or 20 μm Lalistat 2 or Orlistat was added. Free ^14^C- or ^3^H-fatty acids in CE and TG hydrolase assays were determined by scintillation counting. Released ROH in RE hydrolase assay was determined by HPLC-FD. Data are presented as mean ± S.D. for duplicate determinations (*n* = 4 for all groups). Statistically significant differences were determined by Student's unpaired *t* test (two tailed: *, *p* < 0.05; **, *p* < 0.01; ***, *p* < 0.001). *n.d.* = not detectable.

Lipa^tm1a^ mice, carrying the targeted mutation 1a allele, which harbors reporter and selection genes and the floxed *Lipa* exon 4, were crossed with FLP-expressing mice to remove the reporter and resistance genes (Fig. 1*A*). Offspring carrying the floxed Lipa allele were crossed with mice expressing Cre recombinase under the control of the albumin promoter, which resulted in mice lacking LAL expression specifically in hepatocytes (hep-LAL-ko, Fig. 1*A*). To confirm the hepatocyte-specific LAL knockout, we isolated primary hepatocytes and nonparenchymal cells (NPCs). Purities of the cell preparations were confirmed by high-mRNA expression levels of the hepatocyte markers glucose-6-phosphatase (*G6Pase*) and glycerol kinase (*GK*) in hepatocytes and high mRNA expression of the endothelial cell marker *CD31* as well as macrophage marker *F4/80* in NPCs (Fig. 1*B*). Analysis of *Lipa* mRNA levels indicated >96% decreased *Lipa* expression levels in total liver and hepatocytes, whereas the levels in NPC fraction were virtually unchanged, consistent with a hepatocyte-specific deletion of *Lipa* (Fig. 1*C*).

Next, we compared lipolytic activities of isolated WT and hep-LAL-ko hepatocytes by performing *in vitro* activity assays at acidic pH (pH 4.5) using cholesteryl oleate, triolein, and RP as substrates. We observed that irrespective of the substrates, acid hydrolytic activities in lysates of hepatocytes from hep-LAL-ko mice were at least 65% lower compared with WT littermates (Fig. 1*D*). Furthermore, addition of Lalistat 2, a specific LAL inhibitor (12), decreased hydrolytic activities of WT lysates to levels similar to that of hep-LAL-ko lysates, whereas the addition of Orlistat, a more general serine hydrolase inhibitor (13), further reduced acid hydrolytic activities of both WT and hep-LAL-ko lysates (Fig. 1*D*). Interestingly, addition of Lalistat 2 to lysates prepared from hepatocytes of hep-LAL-ko mice decreased TG and RE hydrolase activities by 30 and 50%, respectively. Together, results indicate that hepatocyte-specific deletion of LAL leads to a pronounced reduction of acid neutral lipid ester hydrolase activities. The presence of Lalistat 2–inhibitable activities in lysates of hep-LAL-ko hepatocytes indicates the presence of the LAL protein, which may derive from impurities by other liver cell types and/or extracellular uptake.

### hep-LAL-ko mice specifically accumulate CE but not TG and RE in the liver

**Figure 2.**
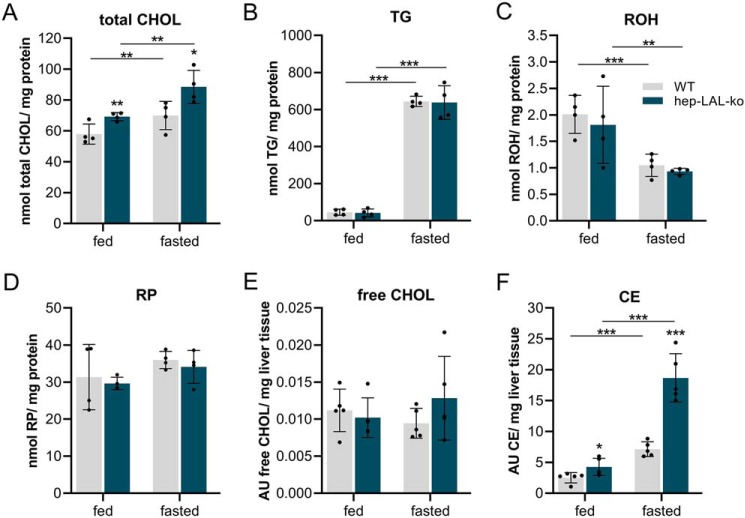
**Mice lacking LAL specifically in hepatocytes accumulate cholesteryl ester in the liver.** Liver was collected from *ad libitum*-fed and overnight-fasted male hep-LAL-ko mice and littermates (*WT*) at an age of 5 months (*A–D*) or 3 months (*E* and *F*). Lipids were Folch-extracted and total CHOL (*A*) and TG (*B*) were determined by commercial kits. *C,* ROH and RP were *n*-hexane–extracted and analyzed by HPLC-FD. CHOL (*E*) and CE (*F*) were Folch-extracted and analyzed by QQQ/MS. Amounts of neutral lipids were normalized to milligrams of protein or milligrams of tissue. Data are presented as mean ± S.D. for duplicate determinations (*n* = 4 for all groups). Statistically significant differences were determined between genotypes and feeding status by Student's unpaired *t* test (two-tailed; *, *p* < 0.05; **, *p* < 0.01; ***, *p* < 0.001).

To investigate whether reduced acid neutral lipid ester hydrolase activities in hep-LAL-ko hepatocytes led to respective lipid accumulation, we analyzed neutral lipid content in the livers of fed and fasted hep-LAL-ko mice and WT littermates (Fig. 2). Compared with WT, the livers of fed and fasted hep-LAL-ko mice contained 16 and 21% increased total CHOL levels, respectively (Fig. 2*A*). Interestingly, hepatic TG, ROH, and RP levels were not different between genotypes, irrespective of the feeding status of the animals (Fig. 2, *B–D*). To differentiate whether free and/or esterified CHOL (= CE) were increased in the livers of hep-LAL-ko mice, we performed QQQ/MS measurements. We found that free CHOL levels in the livers of fed and fasted mice were not different between genotypes, whereas the amounts of CE were 1.7 and 2.6 times higher in livers of fed and fasted hep-LAL-ko, respectively, as compared with WT littermates (Fig. 2, *E* and *F*).

Next, we investigated whether increased hepatic CE levels of hep-LAL-ko mice affected plasma lipid or glycerol levels of fed or fasted mice. We found that irrespective of the feeding status, hep-LAL-ko mice and WT littermates exhibited comparable levels of investigated parameters (Fig. S1, *B–F*), with the exception of a 12% increase in total CHOL levels in hep-LAL-ko mice in the fasted state (Fig. S1*A*).

Together, measurements of hepatic lipid levels suggest that under standard chow diet, independent of the feeding regimens (fed/fasted), LAL is limiting for CE but not for TG and RE turnover.

### Hep-LAL-ko mice are not impaired in plasma lipid clearance

**Figure 3.**
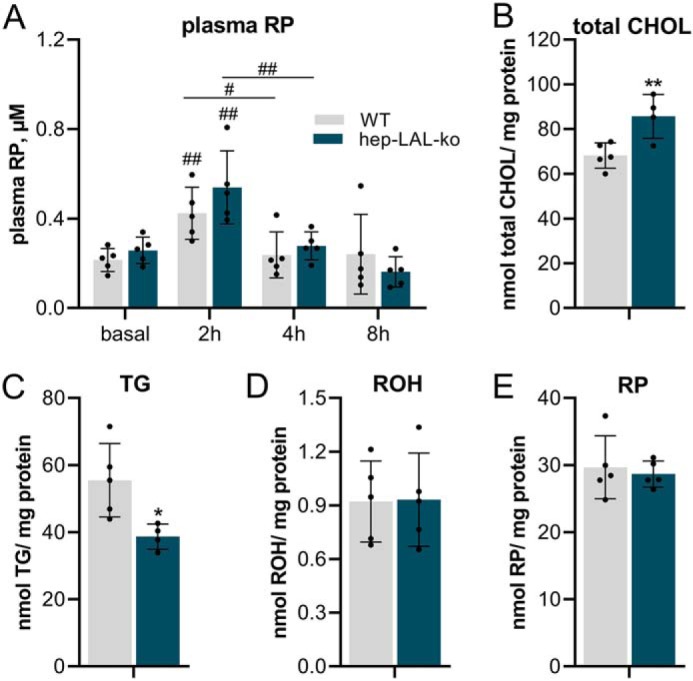
**Bolus administration of vitamin A/cholesterol/olive oil gavage to mice lacking LAL specifically in hepatocyte decreases hepatic triglyceride levels.** Male hep-LAL-ko mice and littermates (WT) at an age of 5 months received a vitamin A/cholesterol/olive oil gavage (100 μg of retinol/200 μg of cholesterol in 100 μl of olive oil/mouse). Blood was drawn before (= basal, *ad libitum*-fed) and 2, 4, and 8 h after gavage and *n*-hexane–extracted for RP analyses by HPLC-FD (*A*). Liver was collected after 8 h of gavage and homogenized. Lipids were Folch-extracted, and total CHOL (*B*) and TG (*C*) were determined by commercial kits. ROH (*D*) and RP (*E*) were *n*-hexane–extracted and determined by HPLC-FD. Hepatic neutral lipid content was normalized to milligrams of protein. Data are presented as means ± S.D. for duplicate determinations (*n* = 5 for all groups) and representative for two independent experiments. Statistically significant differences were determined between genotypes and different time points by Student's unpaired *t* test (two tailed: #, *p* < 0.05; ##, *p* < 0.01 between time points; *, *p* < 0.05; **, *p* < 0.01 between groups).

Because hepatocyte-specific deletion of LAL may affect plasma lipid clearance and hepatic lipid turnover, we challenged hep-LAL-ko mice with a VitA/CHOL/olive oil gavage. Two hours after lipid bolus administration, plasma RP levels were similarly increased in hep-LAL-ko mice and WT littermates and were back to basal levels after 4 and 8 h (Fig. 3*A*). The similar decrease in plasma RP levels 4 h after lipid bolus administration indicated that plasma lipid clearance of hep-LAL-ko mice was not compromised. Eight hours after lipid bolus administration, we analyzed the neutral lipid content of the liver. As expected, the livers of hep-LAL-ko mice contained 20% higher total CHOL levels (Fig. 3*B*). Remarkably, however, livers of hep-LAL-ko mice contained 30% lowered amounts of TG as compared with WT littermates (Fig. 3*C*). In contrast, no differences in hepatic ROH and RP contents were observed between genotypes (Fig. 3, *D* and *E*).

### hep-LAL-ko mice exhibit lower hepatic TG and RE levels in response to VitA/HFD feeding

**Figure 4.**
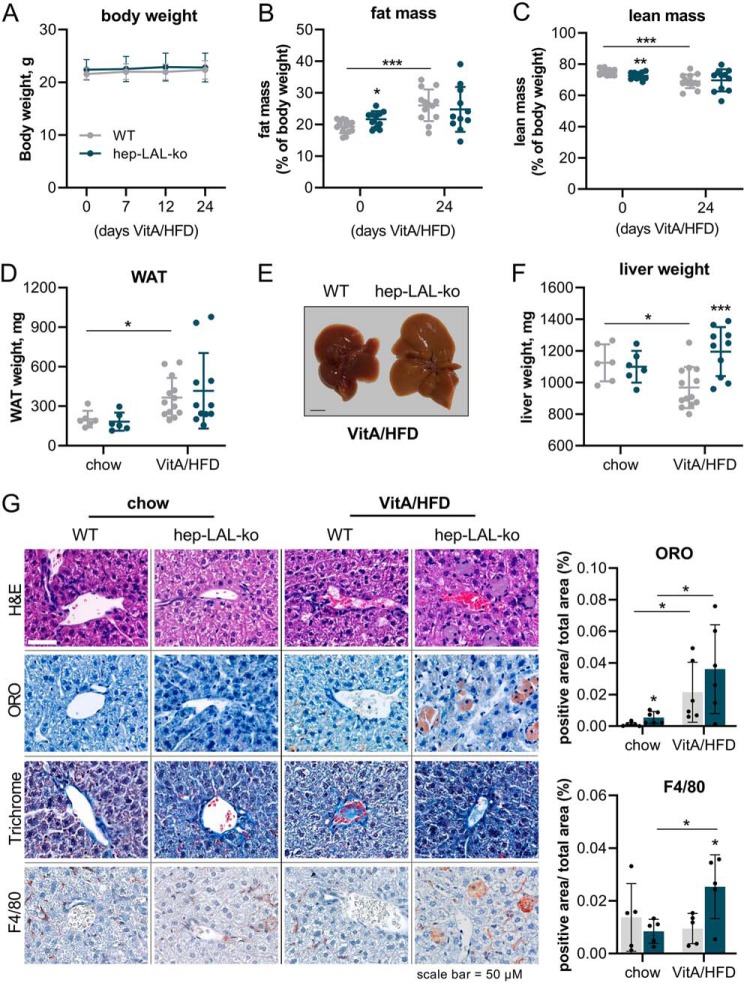
**Vitamin A excess/HFD feeding of hepatocyte-specific LAL-deficient mice induces hepatomegaly and increased numbers of F4/80-positive cells in liver sections.** Female hep-LAL-ko mice and littermates (*WT*) at an age of 5 months were fed a standard chow or a vitamin A excess (100,000 IU vitamin A)/HFD for 3 weeks. *A,* weekly measurements of body weight. Fat mass (*B*) and lean mass (*C*) were determined by NMR analyses before (day 0) and after feeding a VitA/HFD (day 21) and are expressed as % of body weight. *D,* WAT weights. *E,* macroscopic depiction of excised liver of *ad libitum*-fed mice on VitA/HFD. *Scale bar* represents 1 cm. *F,* liver weights. *G,* liver sections of standard chow or VitA/HFD-fed hep-LAL-ko mice and WT littermates were stained with H&E (cell morphology), ORO (neutral lipids), CAB trichrome (collagen), or incubated with antibody against F4/80 (macrophages, Kupffer cells). Hematoxylin was used for counterstaining of nuclei. Representative images (*n* = 6) are depicted. Quantification of ORO- and F4/80-positive areas was performed using IHC Toolbox Plugin for ImageJ software (46) and normalized to total inspected area. Data are presented as mean ± S.D. for duplicate determinations (*n* = 11 for hep-LAL-ko and *n* = 13 for WT on VitA/HFD, and *n* = 6 for both genotypes on chow diet). Statistically significant differences were determined between genotypes and treatment by Student's unpaired *t* test (two tailed: *, *p* < 0.05; **, *p* < 0.01; ***, *p* < 0.001).

**Figure 5.**
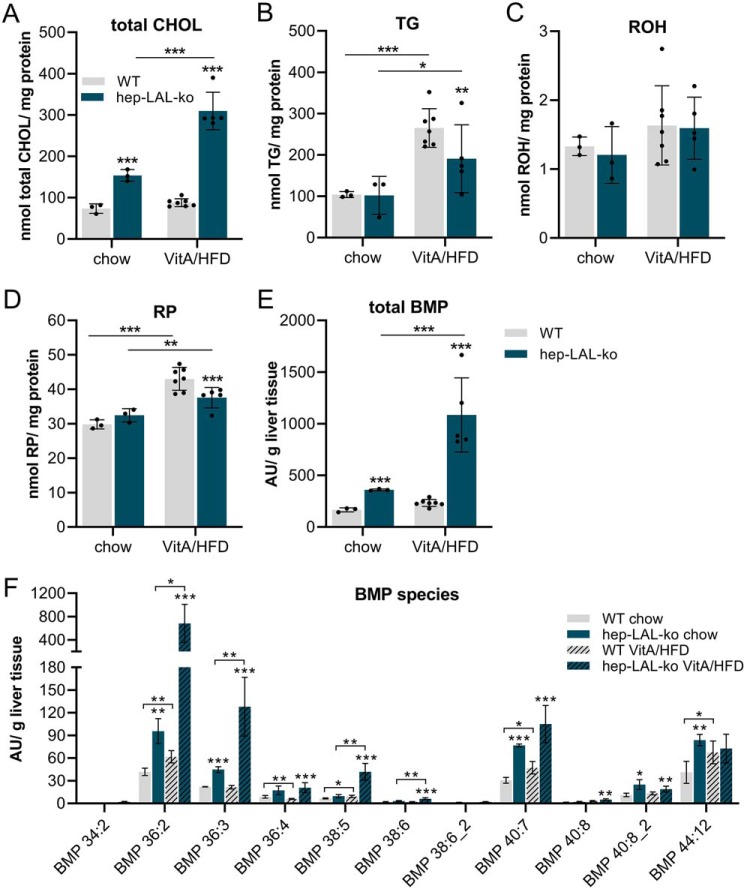
**Vitamin A excess/HFD feeding of hepatocyte-specific LAL-deficient mice leads to increased hepatic cholesteryl ester and bis(monoacylglycero)phosphate but lower triglyceride content.** Female hep-LAL-ko mice and littermates (WT) at an age of 5 months were fed a standard chow or a vitamin A excess (100,000 IU VitA)/HFD for 3 weeks. Lipids of liver homogenates were Folch-extracted and total CHOL (*A*) and TG (*B*) were determined by commercial kits. For ROH (*C*) and RP (*D*) measurements, liver lipids were *n*-hexane–extracted, and retinoids were determined by HPLC-FD. For total BMP (*E*) and BMP species (*F*), liver homogenates were Folch-extracted and analyzed by QQQ/MS. Amounts of lipids were normalized to milligrams of protein or grams of tissue. Data are presented as mean ± S.D. for duplicate determinations (*n* = 5–11 for hep-LAL-ko and *n* = 7–13 for WT on VitA/HFD, and *n* = 3–6 for both genotypes on chow diet). Statistically significant differences were determined between genotypes and treatment by Student's unpaired *t* test (two tailed: *, *p* < 0.05; **, *p* < 0.01; ***, *p* < 0.001).

The observation of lower hepatic TG levels upon lipid bolus administration prompted us to challenge hep-LAL-ko mice with a chronic VitA/HFD feeding regimen, which has increased VitA, CHOL, and fat content and thus may induce an even more pronounced deregulation of hepatic neutral lipid turnover. Feeding a VitA/HFD for 21 days did not induce changes in body weight of hep-LAL-ko mice and WT littermates (Fig. 4*A*), presumably because of the relatively short duration and/or the advanced age of the animals. VitA/HFD feeding induced increased fat mass (∼35%) and decreased lean mass (∼8%) in WT mice and a similar trend toward increased fat mass and decreased lean mass in hep-LAL-ko mice (Fig. 4, *B* and *C*). Consistent with increased fat mass, white adipose tissue (WAT) weights in mice of both genotypes were increased (>1.8-fold, Fig. 4*D*). Macroscopic evaluation showed an enlargement and a more yellowish color of the liver from hep-LAL-ko as compared with WT littermates (Fig. 4*E*). Accordingly, liver weights of hep-LAL-ko mice on VitA/HFD were ∼20% higher than that of WT littermates (Fig. 4*F*). Histological analyses revealed remarkable morphological changes in liver sections of VitA/HFD fed hep-LAL-ko mice, showing enlarged cells with foamy cytoplasm (H&E stain),which stained positive for the murine macrophage/Kupffer cell marker F4/80 and neutral lipids (Oil Red O, ORO) (Fig. 4*G*). Liver sections from mice of both genotypes fed a VitA/HFD diet showed increased ORO-positive areas of (Fig. 4*G*, *ORO*). Yet, only liver sections of hep-LAL-ko mice showed increased F4/80 positive areas (Fig. 4*G*, *F4/80*). Chromotrop-aniline blue (CAB) trichrome stain of liver sections was not different between the genotype and feeding regimes (Fig. 4*G*, *3rd row*). Lipid analyses of liver from mice fed a standard chow or a VitA/HFD confirmed increased neutral lipid content in livers of VitA/HFD fed hep-LAL-ko mice, because total CHOL content of liver from hep-LAL-ko mice was 2.6-fold higher compared with WT liver (Fig. 5*A*). Unexpectedly however, hepatic TG and RP of these mice were 33 and 13% lower, respectively, as compared with WT littermates (Fig. 5, *B* and *D*). No changes in hepatic ROH levels were observed between genotypes (Fig. 4*C*). Next, we analyzed hepatic phospholipid composition. Interestingly, we observed a 2-fold increased total BMP level of hep-LAL-ko mice on standard chow diet, which was even more elevated in liver of VitA/HFD fed hep-LAL-ko mice (3.5-fold compared with WT, Fig. 5*E*). Analysis of various BMP species showed the highest increase in BMP 36:2 (10-fold, Fig. 5*F*). Compared with increased hepatic BMP levels, the contents of other phospholipids, including PC, PI, SM, LPC, LPI, and LPE, were unchanged or decreased (<45%) in liver of VitA/HFD fed hep-LAL-ko mice (Fig. S2, *A–F*). Next, we investigated whether the liver pathology translated into changes in plasma lipid (total CHOL, TG, nonesterified fatty acid, and ROH), glycerol, and ketone body levels. Hep-LAL-ko mice fed a chow or VitA/HFD exhibited no differences in all measured plasma levels as compared with WT littermates, with the exception of 22% higher plasma ROH levels of VitA/HFD fed hep-LAL-ko mice (Fig. S3, *A–F*).

In summary, the observation of increased hepatic CHOL and BMP levels upon VitA/HFD feeding of hep-LAL-ko mice is consistent with the pathology of defective lysosomal storage diseases (14). Enlarged F4/80 and Oil Red O–positive cells in liver sections of VitA/HFD fed hep-LAL-ko mice argue for a critical role of Kupffer cells in the etiopathology, an abnormality also observed in livers of mice globally lacking LAL (6). The observation, however, that feeding hep-LAL-ko mice a VitA/HFD diet led to lower hepatic TG and RE levels suggests that (i) the expression levels of genes involved in hepatic TG and RE synthesis and/or FA utilization are deregulated, leading to decreased TG and RE biosynthesis and/or enhanced FA utilization, and/or that (ii) lysosomal degradation of these lipid esters is catalyzed by other lipases. Alternatively, but a less likely scenario, a small amount of LAL protein, as a result of extracellular uptake, could be present in hepatocytes and sufficient for lysosomal TG and RE clearance but insufficient for CE clearance.

### Hepatocyte-specific deletion of LAL causes decreased expression of genes involved in TG synthesis

**Figure 6.**
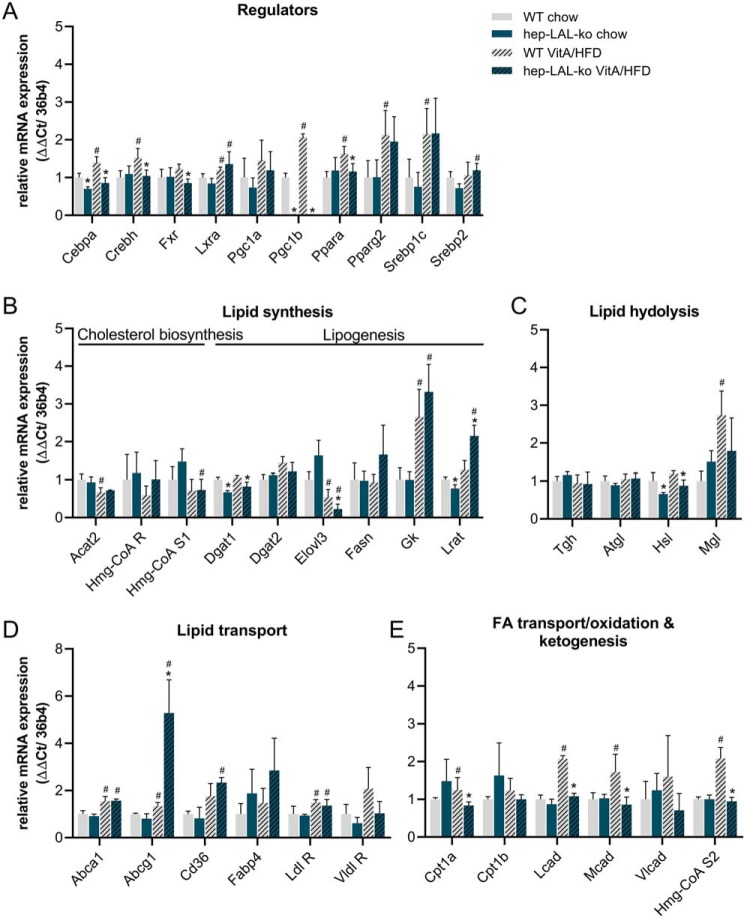
**Hepatocyte-specific deletion of LAL does not induce major changes in hepatic gene expression levels.** Female hep-LAL-ko mice and littermates (WT) at an age of 5 months were fed a standard chow or vitamin A excess (100,000 IU VitA)/HFD for 3 weeks. mRNA from liver was isolated and transcribed into cDNA, and qPCR was performed for transcription factors regulating lipid homeostasis (*A*), acyltransferases of neutral lipid ester synthesis (*B*), lipolytic enzymes (*C*), proteins involved in lipid transport (*D*), and fatty acid oxidation (*E*). Expression levels were calculated by the ΔΔ*Ct* method using *36b4* as ribosomal housekeeping gene and normalized to WT mice on chow diet. Data are presented as mean ± S.D. for duplicate determinations (*n* = 5 for hep-LAL-ko and *n* = 7 for WT on VitA/HFD and *n* = 3 of both genotypes for control groups). Statistically significant differences were determined between genotypes and treatment by Student's unpaired *t* test (two-tailed; *, *p* < 0.05 between groups; #, *p* < 0.05 between feeding regimens). For abbreviations see text and Table S2.

To delineate factors causative for decreased hepatic TG and RP levels, we determined mRNA expression levels of genes involved in lipid homeostasis. Consistent with decreased TG levels in livers of hep-LAL-ko mice fed a VitA/HFD, the mRNA expression levels of several transcription factors inducing TG synthesis (*Cebpa, Crebh, Fxr,* and *Pgc1b*) as well as of the FA elongase *Elovl3* and the acyltransferase *Dgat1*, were decreased by 39, 32, 30, 99, 59, and 23%, respectively (Fig. 6, *A* and *B*). The expression levels of other regulators of TG synthesis (*Srebp1c, Pparg2,* and *Lxra*) were not different between genotypes. Furthermore, in livers of hep-LAL-ko mice on VitA/HFD, mRNA expression levels of genes encoding the lipase *Hsl*, the transcription factor *Ppara*, and several mitochondrial proteins involved in FA transfer, β-oxidation, and ketogenesis (*Cpt1a, Lcad, Mcad,* and *Hmg-CoA S2*) were 27, 30, 23, 48, 51, and 54% lower, respectively (Fig. 6, *A* and *E*). Reduced mRNA expression levels of several mitochondrial genes in livers of hep-LAL-ko mice fed a VitA/HFD were accompanied by decreased mitochondrial DNA content (Fig. S4*C*). Interestingly, however, this did not translate into mitochondrial protein content nor function. Protein expression levels of mitochondrial marker proteins NADH:ubiquinone oxidoreductase core subunit S1 (NDUFS1) and mitochondrially-encoded cytochrome *c* oxidase I (MT-CO1) were similar in liver homogenates obtained from hep-LAL-ko mice and WT littermates. Furthermore, analyses of mitochondrially-enriched liver fractions from hep-LAL-ko mice showed similar protein expression levels of carnitine *O-*palmitoyltransferase 1A (CPT1A), the rate-limiting enzyme of FA β-oxidation, which corresponded with unchanged rates of trapped CO_2_ and acid-soluble metabolites as compared with WT littermates (Fig. S4, *A, B,* and *D*).

Together, the decreased hepatic TG content of hep-LAL-ko mice might be a result of lower hepatic expression levels of several regulators (*Cebpa, Crebh,* and *Fxr*) and enzymes (*Elovl3* and *Dgat1*) involved in TG biosynthesis. However, unchanged mRNA expression levels of master regulators of TG and FA synthesis (*Srebp1c* and *Pparg2*) speak against this assumption. Thus, we speculated that other mechanisms may contribute to reduced hepatic TG and RE levels of hep-LAL-ko mice fed a VitA/HFD.

### Mice globally lacking LAL exhibit residual acid hydrolytic activity against TG and RE

The hepatic lipid phenotype of hep-LAL-ko mice suggests that LAL is limiting for the hydrolysis of CE and that additional enzymes for the hydrolysis of TG and RE exist. For the investigation of non-LAL acid hydrolytic activities, we used liver tissues from an LAL-deleter strain (LAL-del), because these tissues are completely devoid of LAL expression. LAL-del mice had been generated by breeding mice carrying the Lipa^tm1a^ allele with mice expressing Cre recombinase under the control of the *CMV* promoter (Fig. S5*A*). These mice globally lack *Lipa* expression (Fig. S5*B*). The functional knockout of LAL was evident by the very pronounced liver phenotype (7, 15), as liver macroscopically appeared severely enlarged and yellowish in color (Fig. S5*C*).

**Figure 7.**
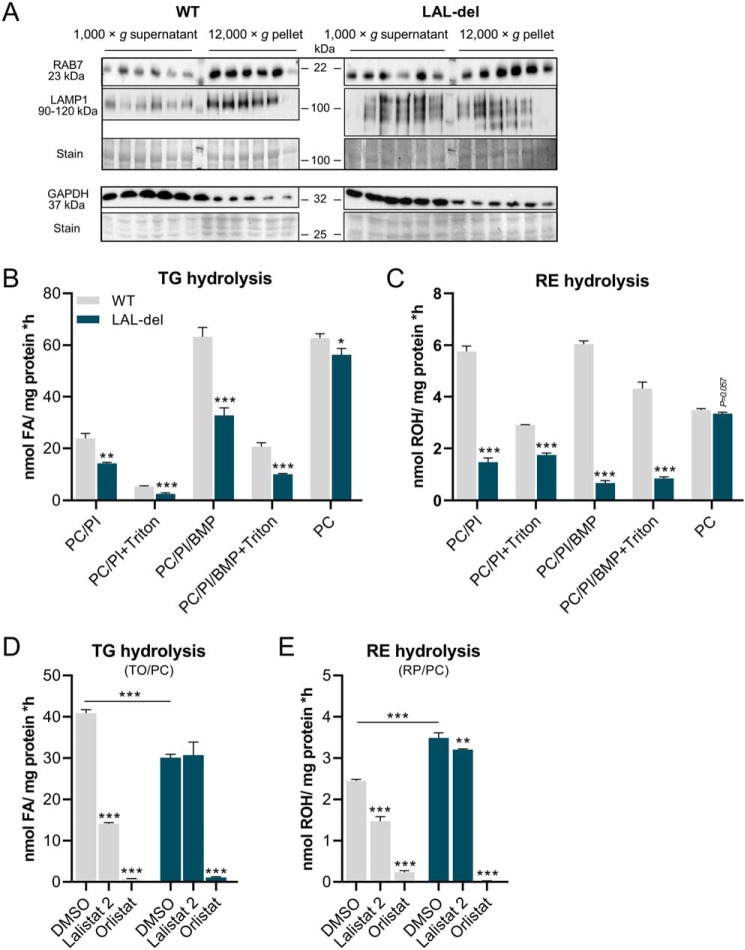
**Determinations of remnant acid hydrolase activity in lysosome-enriched liver fractions.** Liver of 3-month-old female LAL deleter mice (*LAL-del*, globally lacking LAL expression) and littermates (WT) were homogenized, and 1,000 × *g* supernatant and 12,000 × *g* pellet (lysosome-enriched fraction) were prepared. *A, upper blot:* Western blotting of 1,000 × *g* supernatant and 12,000 × *g* pellet proteins probed with antibodies specific for late endosome marker Ras-related protein 7 (RAB7) and late endosome/lysosome marker lysosome-associated membrane protein 1 (LAMP1). Coomassie Blue stain was used as loading control. *Lower blot:* same procedure as for *upper blot*. Western blotting was probed with antibody specific for cytosolic marker protein GAPDH. *B–E*, lysosome-enriched fraction was used for acidic TG and RE hydrolase assays. If indicated, 0.1% Triton X-100 (final concentration) was added to the lysates. *B* and *C*, substrates contained either 300 μm [^3^H]TO or RP, respectively, emulsified with PI and/or PC, and in some cases BMP in sodium acetate buffer (pH 4.5) including FA-free BSA. TG hydrolase assay using TO/PC (*D*) and RE hydrolase assay (*E*) using RP/PC were emulsified in sodium acetate buffer (pH 4.5), including FA-free BSA, ± 20 μm Lalistat 2, 20 μm Orlistat, or solvent DMSO, respectively. The release of ^3^H-FAs in TG hydrolase assays or ROH in RE hydrolase assays was determined by scintillation counting or HPLC-FD, respectively. Data are presented as mean ± S.D. for duplicate determinations (*n* = 6 for both LAL-del and WT). Statistically significant differences were determined between genotypes and treatment by Student's unpaired *t* test (two tailed: *, *p* < 0.05; **, *p* < 0.01; ***, *p* < 0.001).

To explore remnant, non-LAL acid hydrolytic activities for neutral lipid esters, we prepared homogenates from the livers of LAL-del mice and WT littermates and prepared a crude lysosome-enriched fraction by centrifugation at 12,000 × *g*. Western blotting analyses of the 12,000 × *g* pellet from liver homogenates of WT mice showed higher band intensities for the late endosomal/lysosomal marker proteins RAB7 and LAMP1 and lower band intensities for the cytosolic marker protein GAPDH as compared with the 1,000 × *g* supernatants (Fig. 7*A*). This pattern of band intensities was consistent with an enrichment of endosomes/lysosomes in the 12,000 × *g* pellet. Similar patterns were observed in Western blotting analyses of liver fractions from LAL-del mice. However, band intensities of RAB7 and LAMP1 were more comparable between the 12,000 × *g* pellet fractions *versus* 1,000 × *g* supernatants, presumably due to the higher neutral lipid content (6, 16), which renders separation by differential centrifugation challenging. Then, we subjected the lysosome-enriched liver fractions of WT and LAL-del mice to TG and RE hydrolase activity assays using triolein and RP as substrates. LAL is known to exert little activity when the substrate is emulsified with PC but is activated by negatively charged phospholipids such as BMP (9, 17). Thus, we emulsified the substrate with different combinations of uncharged and negatively charged phospholipids. As expected, the relative differences in the acid hydrolytic activities of lysosome-enriched fractions from WT liver *versus* LAL-del liver for TG and RE as substrates were highest when the substrates were emulsified in the presence of the negatively charged phospholipids PC/PI or PC/PI/BMP (Fig. 7, *B* and *C*). Addition of the detergent Triton X-100 decreased hydrolytic activities against TG and RE in a similar manner, but in relative terms, acid hydrolytic activities of lysosome-enriched fractions from WT liver were always significantly higher than those from LAL-del liver (Fig. 7, *B* and *C*). Remarkably, when we emulsified the TG and RE substrate with PC alone, we observed that the acid hydrolytic activities of lysosome-enriched liver fractions from WT and LAL-del mice were almost identical (Fig. 7, *B* and *C*), indicating that additional acid hydrolases must exist that are active in the absence of negatively charged phospholipids. To further explore the relative contribution of LAL and other lipases to acid hydrolytic activities of PC-emulsified TG and RE substrates, we added Lalistat 2 and Orlistat to the assay mixtures. Addition of Lalistat 2 decreased acid hydrolytic activities of lysosome-enriched fractions from WT liver for TG and RE substrates by 75 and 40%, respectively (Fig. 7, *D* and *E*). As expected, addition of Lalistat 2 had no effect on acid TG hydrolytic activities of lysosome-enriched fractions of LAL-del liver, but it decreased acid RE hydrolytic activities by 9% (Fig. 7, *D* and *E*). Addition of Orlistat to the assay mixtures virtually blunted acid hydrolytic activities, irrespective of the substrates and genotypes of mice used for lysosome-enriched liver fractions (Fig. 7, *D* and *E*).

Together, these results show that, in addition to LAL, acid hydrolytic activity for TG and RE exists that can be inhibited by Orlistat. Apparently, these hydrolytic activities are distinct to LAL activity because they (i) exist in LAL-del liver tissue, (ii) are not sensitive to Lalistat 2, and (iii) are not stimulated by negatively charged phospholipids.

### Remnant acid TG hydrolase activity is up-regulated in livers of hep-LAL-ko mice on VitA/HFD

**Figure 8.**
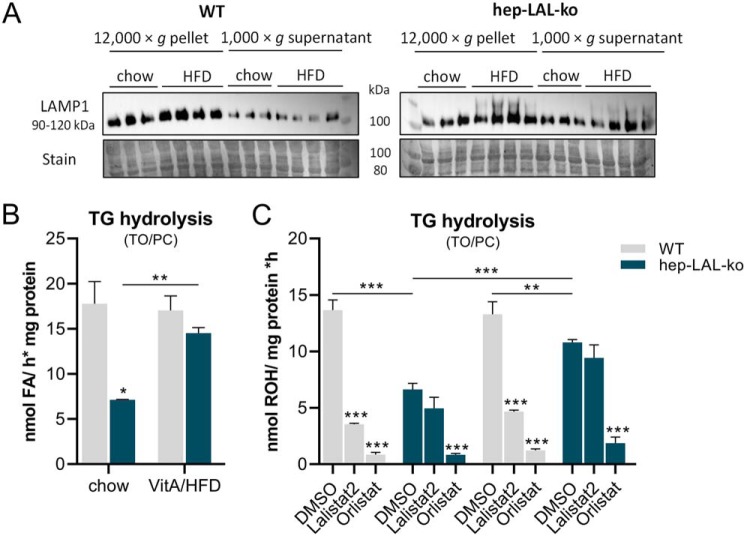
**Feeding a vitamin A excess/HFD increases acid triglyceride hydrolase activity in lysosome-enriched liver fractions of hepatocyte-specific LAL-deficient mice.** Female hep-LAL-ko mice and littermates (WT) at an age of 5 months were fed *ad libitum* a vitamin A excess (100,000 IU VitA)/HFD for 3 weeks. Age- and gender-matched control groups (*hep-LAL-ko* and *WT*) were fed *ad libitum* a standard chow diet. Liver was homogenized, and a 1,000 × *g* supernatant and 12,000 × *g* pellet (lysosome-enriched fraction) were prepared. *A,* Western blot analysis of 1,000 × *g* supernatant and 12,000 × *g* pellet using antibody specific against late endosome/lysosome marker lysosome-associated membrane protein 1 (LAMP1). Coomassie Blue stain was used as loading control. *B* and *C*, lysosome-enriched fraction was incubated with 300 μm TO, 10 μCi/ml [^3^H]TO, and 45 μm PC in 100 μm sodium acetate buffer (pH 4.5), including FA-free BSA. In some cases, 20 μm Lalistat 2, 20 μm Orlistat, or DMSO as solvent was added. The release of ^3^H-FAs in TG hydrolase assays was determined by scintillation counting. Data are presented as mean ± S.D. for duplicate determinations (*n* = 4 for hep-LAL-ko and *n* = 4 for WT on VitA/HFD and *n* = 3 of both genotypes for control groups) and are representative for two independent experiments. Statistically significant differences were determined between genotypes and treatment by Student's unpaired *t* test (two tailed: *, *p* < 0.05; **, *p* < 0.01; ***, *p* < 0.001).

The observation that the livers of hep-LAL-ko mice on VitA/HFD contained decreased amounts of TG led us to hypothesize that this might be the consequence of an up-regulation of acid non-LAL TG hydrolase activity. Thus, we prepared lysosome-enriched fractions (12,000 × *g* pellet) from livers of WT and hep-LAL-ko mice. Western blotting analyses showed LAMP1 enrichment in the lysosome-enriched fractions *versus* 1,000 × *g* supernatant from livers of WT mice. Differences in the LAMP1 band intensities of lysosome-enriched fractions *versus* 1,000 × *g* supernatant from livers of hep-LAL-ko mice were similarly subtle as observed in preparations from livers of LAL-del mice (Fig. 8*A*, compare with 7*A*). For acid TG hydrolase activity assays, we emulsified triolein substrate with PC alone to favor remnant, non-LAL hydrolytic activities. Lysosome-enriched fractions from livers of hep-LAL-ko mice contained 40% lower acid TG hydrolase activity as compared with preparations of WT mice (Fig. 8*B*). Interestingly, feeding hep-LAL-ko mice a VitA/HFD diet for 3 weeks led to an increase in acid TG hydrolase activities of lysosome-enriched liver fractions to similar levels as in preparations of WT mice (Fig. 8*B*). Because this increased acid TG hydrolase activity could be a result of *e.g.* infiltration of LAL-expressing cells in livers of hep-LAL-ko mice when fed a VitA/HFD diet, we performed similar activity assays in the presence of the inhibitors Lalistat 2 and Orlistat. Although addition of Lalistat 2 inhibited acid TG hydrolase activity by more than 65% in lysosome-enriched fractions from livers of WT mice of both dietary regimens, no statistically significant inhibition was observed in preparations from hep-LAL-ko mice (Fig. 8*C*). Addition of Orlistat to the assay mixtures containing lysosome-enriched fractions from mice of both genotypes and dietary regimens inhibited acid TG hydrolase activities in all cases by at least 82%.

In summary, these data indicate that acid TG hydrolase activity in livers of hep-LAL-ko mice on VitA/HFD is elevated, which may explain lower hepatic TG levels.

## Discussion

In this study, we explored the functional role of LAL in neutral lipid metabolism in hepatocytes. Because LAL exhibits hydrolytic activities against CE, TG, and RE (9), this enzyme is thought to be important for the lysosomal clearance of all these lipid esters. Yet, the diverging neutral lipid ester phenotype of mice globally lacking LAL questions this concept. These mice exhibit massively increased hepatic CE and to a lesser extent TG, but reduced RE levels (6, 9). The observation that global LAL-ko mice exhibit whole-body neutral lipid derangement (8) renders this mouse model inappropriate to address this question. To avoid such severely deranged neutral lipid homeostasis, we used a mouse model lacking LAL specifically in hepatocytes, limiting defective lysosomal clearance of neutral lipid esters to the liver and particularly to hepatocytes, which are considered the key site for dietary lipid turnover. In this study, we observed that hep-LAL-ko mice showed a relatively mild CE accumulation phenotype in the liver (up to a 2-fold increase in CE compared with a 50-fold increase in hepatic CE content of LAL-ko mice (9)). Unexpectedly, hepatic TG and RE levels remained unchanged. Administration of a single VitA/CHOL/olive oil gavage or feeding a VitA/HFD diet for 3 weeks, as an acute and chronic challenge for hepatic neutral lipid turnover, respectively, did not induce hepatic TG and/or RE accumulation. In contrast, the hepatic TG and RE levels of hep-LAL-ko mice were even decreased. Reduced hepatic TG levels upon VitA/HFD feeding might be a consequence of increased acid TG hydrolase activity and/or decreased lipid synthesis.

The CE accumulation in livers of hep-LAL-ko mice clearly demonstrates a limiting role of LAL for the lysosomal clearance of CE. This was expected because mice deficient in LAL activity (6, 7, 9) as well as humans suffering CESD or Wolman disease (18) exhibit a very pronounced CHOL accumulation phenotype in the liver and other tissues. Furthermore, histological analyses in this study pointed toward a very specific role of Kupffer cells in the etiopathology. Staining of liver sections from hep-LAL-ko mice shows enlarged lipid-loaded Kupffer cells that have also been observed in mice and humans lacking LAL activity (6, 19). Yet, the severity of histological changes and the magnitude of CE accumulation in liver of hep-LAL-ko mice, even when challenged with a VitA/HFD diet, was relatively minor. A possible explanation for this relatively mild CE accumulation phenotype could be that other liver cell types contribute significantly to the overall uptake and clearance of chylomicron remnants so that a larger proportion of chylomicron-associated CE is actually processed in NPCs. However, this scenario is not very likely because radiolabeled chylomicron remnant injection studies found that the majority of radioactivity (65–80%) appeared in the parenchymal fraction (20–22). A more plausible explanation is that considerable amounts of LAL protein are secreted by various cell types, such as endothelial cells, fibroblasts, and smooth muscle cells (23, 24), and is taken up from other cells such as hepatocytes (25). Therefore, hepatocytes of hep-LAL-ko mice, although they are not expressing LAL themselves, contain a certain amount of LAL activity in their lysosomes. Experimentally, the uptake of circulating LAL protein has been demonstrated in mice and humans (26–30). LAL-ko mice treated with *Pichia*, plant, or CHO cell-expressed human recombinant LAL showed uptake into liver/hepatocytes/Kupffer cells and normalization of hepatic color and decreased hepatic CHOL and TG contents (26, 27, 31). Similarly, injection of human recombinant LAL protein in LAL-ko mice dose-dependently extended life span and partially corrected CHOL and TG levels in liver and spleen (32). Furthermore, replacement therapy, infusing Sebelipase alfa, a recombinant human LAL enzyme, in human patients has successfully reduced disease severity, as indicated by decreased plasma alanine aminotransferase/aspartate transaminase levels or decreased liver volume and fat content (33). Furthermore, doxycycline (turn off)-inducible transgenic expression of human LAL in mice globally lacking LAL demonstrated that LAL protein was not only detected in the liver but also in the lung and the spleen, where it largely restored the TG and CE accumulation phenotype (11). Together, these studies clearly demonstrate that tissues take up LAL from the bloodstream, which can restore defective lysosomal clearance. Apart from the enzyme therapy approach and studies in mice, endogenous LAL activity has also been found in human plasma, and low plasma levels have been correlated as a predictor for increased severity of liver disease (34, 35). In our acid CE/TG/RE hydrolase activity assays using lysates of hepatocytes from hep-LAL-ko mice, we additionally observed Lalistat 2–sensitive activities (decreased by 73, 27, 48%, respectively), indicative for the presence of LAL protein (Fig. 1*D*). Thus, we assume that LAL protein has been taken up from the circulation by hepatocytes, which restored to some extent defective lysosomal lipid clearance.

The observation of decreased hepatic TG and RE levels of hep-LAL-ko mice fed a VitA/HFD is rather puzzling. Studies by Harrison *et al.* (36), Blomhoff *et al.* (37), and others (38), using ^3^H-labeled chylomicron REs, suggest that the clearance of RE occurs largely by the endosomal pathway and may not require lysosomes (39). Thus, if TG, similar as RE, would not necessarily require lysosomal clearance, the absence of LAL would not be expected to affect cellular TG/RE levels. Furthermore, acid hydrolytic activities for RE in lysosomal liver fractions have been demonstrated in the past (40, 41), and it has been suggested that lysosomes contain at least two different enzymes, one for CE hydrolysis (termed aCEH and identical to LAL) and another one for RE (aREH and distinct from LAL) (41). Although LAL has been shown to hydrolyze not only CE but also TG and RE (9, 40), the study by Mercier *et al.* (41) and the results of this study point toward the existence of different acid-neutral lipid ester hydrolases. In our acid hydrolase activity assays, using PC for emulsifying TG and RE substrates, we found almost similar levels of acid hydrolase activities against TG and RE in lysosome-enriched fractions from the liver of LAL-del *versus* WT mice that were not inhibited by Lalistat 2 (Fig. 7, *D* and *E*). This demonstrates that non-LAL acid hydrolytic activity exists in liver. Interestingly and similar to our assay system, Mercier *et al.* (41) used egg yolk lecithin for substrate preparation for the identification of the two distinct acid hydrolytic activities. Egg yolk lecithin contains mostly PC (∼80%), much less PE (∼12%), and is almost completely devoid of PI (42, 43). Thus, in their assay system the presence of negatively charged lipids was very limited. Authors concluded from their study (41) that an additional acid hydrolase must exist, which is not inhibited by the addition of CE (41). Taken together, it appears feasible that at least two different acid hydrolytic activities exist. Although LAL activity is stimulated by negatively charged phospholipids, the other hydrolytic activity is insensitive to negatively charged phospholipids. The observation that hep-LAL-ko mice contain increased BMP levels, which is known to stimulate LAL activity (9, 17), could also cause increased acid hydrolytic activity. The finding, however, that the remnant acid hydrolytic activity is insensitive to the inhibitor Lalistat 2 indicates that other enzyme(s) are up-regulated upon VitA/HFD feeding of hep-LAL-ko mice and responsible for observed hydrolytic activity.

We also explored the possibility that changes in the expression levels of regulators for lipid biosynthesis and/or hydrolysis/oxidation might explain decreased hepatic TG and RE levels of hep-LAL-ko mice fed a VitA/HFD. Although VitA/HFD feeding of hep-LAL-ko mice failed to induce hepatic expression levels of the lipid regulators *Cebpa*, *Crebh*, and *Pgc1b*, other regulators known to induce lipid biosynthesis, such as *Pparg2* and *Srebp1c*, were not different from WT. In general (with the exception of *Pgc1b*), changes in the expression levels of lipid regulators were rather small, so the decreased expression of lipid regulators or enzymes involved in lipid synthesis could not/might not explain decreased hepatic TG levels of hep-LAL-ko mice on VitA/HFD. Furthermore, despite the very low expression levels of *Pgc1b*, mitochondrial protein content and FA oxidation rates were similar between genotypes. Thus, FA utilization was apparently not altered and is not expected to affect hepatic TG content.

Mice globally lacking LAL expression have been shown to exhibit increased hepatic CHOL biosynthesis (8). From the unchanged hepatic expression levels of *Acat2, Hmg-CoA R, Hmg-CoA S1*, and *Abcg1* between hep-LAL-ko and WT mice (Fig. 5, *B* and *D*), we reasoned that CHOL biosynthesis is not induced in livers of hep-LAL-ko mice. Thus, we conclude that the hepatic CE accumulation of hep-LAL-ko mice is not further aggravated via elevated CHOL biosynthesis, which might explain the less severe CE accumulation phenotype.

In summary, the hepatic CE accumulation phenotypes of hep-LAL-ko mice confirm the limiting role of LAL in CE hydrolysis. The observation that a non-LAL acid hydrolytic activity for TG exists that is up-regulated upon VitA/HFD feeding may explain why hepatic TG levels of hep-LAL-ko mice are decreased. The identification and characterization of such acid lipase(s) will provide important insights into the endosomal/lysosomal lipid-sorting machinery. In the course of preparing this study, similar results on the phenotypes of mice lacking LAL in hepatocytes were reported by Leopold *et al.* (44), and our report expands the characterization of the remnant, non-LAL acid hydrolytic activity in liver.

## Experimental procedures

### Materials

FA-free BSA, (*S*,*S*)-bis(monoacylglycero)phosphate (BMP), β-carotene, cholesterol, retinol (ROH), retinyl palmitate (RP), retinyl acetate, triolein, cholesteryl oleate, l-α-phosphatidylinositol, and 1,2-dioleoyl-*sn*-glycero-3-phosphocholine were purchased from Sigma. Lalistat 2 was a kind gift from Dr. Paul Helquist (Dept. of Chemistry and Biochemistry, University of Notre Dame, Notre Dame, IN).

### Generation of mice lacking LAL specifically in hepatocytes and globally lacking LAL

Lipa^tm1a(EUCOMM)Hmgu^ mice, carrying the *lacZ*-reporter–tagged targeted mutation 1a LAL^tm1a^ (Fig. 1*A*, *top panel*), were generated using embryonic stem cells from EUCOMM (Helmholtz Zentrum Muenchen GmbH, Muenchen, Germany). The general strategy of the “The European Conditional Mouse Mutagenesis Project” is the targeted insertion of a *Frt*-flanked *lacZ*-reporter and a neo-selection cassette, followed by a floxed exon. This construct results in a so-called “knockout-first” strain, containing a dysfunctional *Lipa* gene. Further breeding strategies with mice expressing FLP or Cre recombinase allow excision of reporter/selection cassette or promoter-dependent deletion of the floxed exon.

For the generation of hep-LAL-ko mice, the *Frt*-flanked reporter and resistance gene (neomycin phosphotransferase) as well as the loxP site-flanked exon 4 of the *Lipa* gene were removed by consecutive breeding steps. Therefore, heterozygous Lipa^tm1a(EUCOMM)Hmgu^ mice were crossed with heterozygous FLP-expressing mice that resulted in the deletion of the *Frt*-flanked sequences of the reporter and resistance genes *lacZ* and neomycin phosphotransferase, respectively, and retaining a floxed exon 4 Lipa allele (Fig. 1*A*, *middle panel*). Heterozygous floxed Lipa^tm1a(EUCOMM)Hmgu^ mice were backcrossed three generations on a C57BL/6J background. For hepatocyte-specific LAL deletion, heterozygous offspring carrying the floxed exon 4 Lipa allele were bred with heterozygous mice expressing Cre recombinase under the control of the albumin promoter (Alb-Cre mice), resulting in the deletion of exon 4 of the *Lipa* gene (Fig. 1*A*, *bottom panel*). Offspring were genotyped by PCR for the presence of the floxed allele and Alb-Cre transgene using the primer pairs LAL-flox-fw/LAL-flox-rv and Alb-Cre-fw/Cre-rv, respectively (Table S1). Offspring, homozygous for the Lipa floxed allele (flox/flox) and carrying the Alb-Cre transgene (Cre/+), are hep-LAL-ko mice. Mice homozygous for the floxed Lipa allele are corresponding WT littermates and were used as controls.

Mice with global deletion of LAL were generated by breeding heterozygous Lipa^tm1a(EUCOMM)Hmgu^ mice with heterozygous mice expressing Cre recombinase under the control of the *CMV* promoter. This resulted in the excision of the neomycin resistance cassette and exon 4 of the *Lipa* gene and the generation of the so-called LAL-deleter allele (Fig. S5*A*). Homozygous mice carrying the Lipa deleter allele were backcrossed three times with C57Bl/6J to establish the LAL-del mouse strain (mice homozygous for Lipa deleter allele). Littermates not carrying the Lipa deleter allele were used as WT controls.

Mice were housed on a regular dark/light cycle (14 h light and 10 h dark) at 22 ± 1 °C in a specific pathogen-free environment and kept *ad libitum* on a standard laboratory chow diet (R/M-H Extrudate, V1126-037, Ssniff Spezialdiäten GmbH, Soest, Germany) unless otherwise indicated. For phenotyping of hep-LAL-ko mice, overnight fasted or *ad libitum* fed male mice, 5 months of age, were used. All animal experiments were approved by the Austrian Federal Ministry for Science, Research, and Economy (protocol numbers GZ: 39/9-4/75 ex 2017/18) and conducted in compliance with the council of Europe Convention (ETS 123).

### Administration of oral gavage and diet studies

Male hep-LAL-ko mice and littermates (WT), 5 months of age, received an intragastric oral gavage of 100 μg of ROH (300 IU VitA) and 200 μg of CHOL dissolved in 100 μl of olive oil using a standard gavage needle. Before (time 0) and 2, 4, and 8 h after gavage administration, blood samples were collected by orbital venous sinus bleeding, and EDTA plasma was prepared.

Female hep-LAL-ko mice and littermates, 5 months of age, were fed *ad libitum* with VitA (100,000 IU) HFD (EF R/M pellets, E15744-034, Ssniff Spezialdiäten GmbH), containing 45 kJ % fat and 175 mg/kg CHOL, for 3 weeks. VitA/HFD was prepared by dissolving 50 mg of β-carotene (83,000 IU) in 15 ml of olive oil and dispersion into 1 kg of HFD.

### Histochemistry and immunohistochemistry

Livers of standard chow and VitA/HFD-fed hep-LAL-ko mice and WT littermates were fixed in 4% neutral-buffered formaldehyde for 48 h and embedded in paraffin. Sections (5 μm) were de-paraffinized and subjected to hematoxylin and eosin (H&E) or CAB trichrome staining, respectively, using standard histological techniques (45). Neutral lipids were stained in cryosections of formaldehyde-fixed, nonembedded tissues using ORO, and nuclei were counter-stained with Mayer's hematoxylin. For macrophage staining, formaldehyde-fixed, paraffin-embedded sections were incubated with anti-mouse F4/80 antibody (1:50; Serotec MCA 497GA; Bio-Rad). Antibody binding was visualized using aminoethyl carbazole substrate chromogen (catalog no. 3464; Dako, Glostrup, Denmark). Stained surface areas were quantified with ImageJ software (46) and normalized to total inspected surface area.

### Isolation of primary hepatocytes and nonparenchymal liver cells by collagenase perfusion and centrifugation

Primary hepatocytes and NPCs of hep-LAL-ko mice and WT littermates (male/female, 2 months of age) were isolated as described previously with some modifications (47). In brief, mice were anesthetized, and the abdomen was surgically opened by a vertical incision. Liver was perfused via the portal vein with Krebs-Henseleit buffer (without Ca^2+^ and SO_4_^2−^) for 5 min, followed by a perfusion with Krebs-Henseleit buffer containing 0.2 mg/ml collagenase type II (Worthington), 2% BSA, and 0.1 mm CaCl_2_ for 10 min. Thereafter, liver was excised and disrupted, and the cell suspension was passed through a gauze, followed by filtration through a 70-μm cell strainer. Hepatocytes were separated from NPCs by centrifugation at 50 × *g* for 3 min at 4 °C. Pelleted hepatocytes were washed twice with PBS and stored at −20 °C. Supernatant containing NPCs was centrifuged at 900 × *g* for 5 min at 4 °C. Pelleted NPCs were washed in PBS, suspended in Dulbecco's modified Eagle's medium (4.5 g/liter glucose; Gibco, Invitrogen), containing 10% fetal calf serum (Sigma) and 100 mg/ml primocin. NPCs were cultivated at 37 °C in humidified air at 80% saturation and 5% CO_2_ for 5 days.

### Preparation of cell lysates, tissue homogenates, and lysosome-enriched fractions

Cell lysates were prepared from a cell suspension in solution A (0.25 m sucrose, 1 mm EDTA, 1 mm DTT, 20 μg/ml leupeptin, 2 μg/ml antipain, and 1 μg/ml pepstatin) by sonication (two times for 10 s, amplitude 10%; Sonoplus ultrasonic homogenizer HD3100, Bandeline Electronic GmbH & Co. KG, Berlin, Germany). Nuclei and unbroken cells were removed by centrifugation at 1,000 × *g* for 5 min at 4 °C, and lysates were stored at −20 °C until further use. Livers (∼150 mg) of *ad libitum-*fed hep-LAL-ko/LAL-del mice and WT littermates were homogenized in solution A using a ball mill (Retsch GmbH, Haan, Germany). Homogenates were centrifuged at 1,000 × *g* and at 4 °C for 10 min, and pellets were discarded, and supernatants, excluding floating fatty layer, were collected and recentrifuged as described above. Resulting supernatants (1,000 × *g* supernatant fraction) were aliquoted or used for preparation of a lysosome-enriched fraction as described (48) with some modifications: Briefly, 1,000 × *g* supernatants were centrifuged at 12,000 × *g* and 4 °C for 30 min. The obtained pellets were resuspended in solution A and homogenized by sonication (two times for 10 s, amplitude 10%) (= lysosome-enriched fraction). Protein concentrations were determined by protein assay (Bio-Rad) according to the manufacturer's instructions using BSA as standard.

### Extraction of neutral lipids for quantitation by HPLC-FD or colorimetry

For retinoid analysis, liver tissues (10–20 mg) were homogenized in 1 ml of *n*-hexane (containing 1 mm butylated hydroxytoluene), 200 μl of H_2_O, and 200 μl of ethanol (containing 1.14 μm all-*trans*-retinyl acetate as internal standard) using a ball mill. Plasma samples (20 μl) were directly *n*-hexane–extracted as described above. Phase separation was obtained by centrifugation at 5,000 × *g* and at 4 °C for 10 min, and upper organic phase was collected. For repeated extraction, 1 ml of *n*-hexane was added to remaining tissue homogenate, vortexed for 30 s, and recentrifuged as above. The organic phases were combined, dried in a speed-vac (Labconco, Kansas City, MO), and stored at −20 °C or immediately used for HPLC fluorescence detection (FD) analysis.

For colorimetric measurements, lipids were extracted from liver tissues (50–70 mg) according to Folch *et al.* (49). Briefly, tissues were homogenized in 1 ml of chloroform/methanol (2:1, v/v) using a ball mill. For phase separation, 200 μl of H_2_O were added, and samples were vortexed for 30 s and centrifuged at 5,000 × *g* at 4 °C for 10 min. Lower organic phase was collected. For repeated extraction, another 500 μl of chloroform were added, mixed by vortexing, and centrifuged as above. The lower organic phases were combined and dried using a speed-vac. Extracts were dissolved in 0.1% Triton X-100 by incubation for 4 h at 37 °C at 500 rpm in a thermomixer, and subsequent sonication was in a water bath sonicator.

Protein determinations were performed by dissolving the dried remaining interphase (*n*-hexane extraction) or infranatant (Folch extraction) in 0.3 n NaOH, 0.1% SDS for 4 h. Protein content was determined by Pierce^TM^ BCA protein assay kit (Thermo Fisher Scientific) according to the manufacturer's instructions using BSA as standard.

### Quantification of retinoids by HPLC-FD

Dried lipid extracts of the *n*-hexane extraction were dissolved in 1 ml of methanol/toluene (1:1, v/v) and separated on a YMC-Pro C18 column (150 × 4.6 mm, S-5 μl, 12 nm, YMC Europe GmbH, Dinslake, Germany) using a gradient solvent system (flow, 1 ml/min; gradient, 1–5 min 100% methanol, 5–14 min 60:40% methanol/toluene, and 14–18 min 100% methanol). Fluorescence was detected at excitation 325 nm/emission 490 nm. The HPLC consisted of a Waters e2695 separation module, including a column oven (at 25 °C) and a Waters 2475 fluorescence detector (Waters). Data were analyzed using Empower 3 chromatography data software (Waters).

### Quantification of TG and total CHOL by colorimetric tests

TG and total CHOL contents were analyzed by commercial triglyceride infinity kit (Thermo Fisher Scientific) and a cholesterol CHOD-PAP kit (Roche Applied Science), respectively. Dissolved lipid extracts were used for measurements according to the manufacturer's instructions.

### Extraction and quantification of CHOL, CE, and phospholipids by UPLC and QQQ/MS analysis

Total lipids of liver explants were extracted twice according to Folch *et al.* (49) using 4 ml of chloroform/methanol (2:1, v/v) containing 500 pmol of butylated hydroxytoluene, 1% acetic acid, and 1 nmol of internal 17:0 CE, 0.5 nmol of 17:0–17:0 PC (Larodan, Solna, Sweden), 0.5 nmol each of 17:1 LPC, d18:1/17:0 SM, 17:0–17:0 PE, 17:1 LPE, 14:0–14:0 BMP (Avanti Polar Lipids, Alabaster, AL), as described previously (50). Dried lipids were dissolved in 500 μl of methanol/2-propanol/water (25:70:10, v/v/v) for UPLC-MS analysis. Chromatographic separation was performed as described (51) with modifications using an AQUITY-UPLC system (Waters) equipped with a Kinetex EVO-C18 column (2.1 × 50 mm, 1.7 μm; Phenomenex), starting a 20-min linear gradient with 80% solvent A (methanol/H_2_O, 1:1, v/v; 10 mm ammonium acetate, 0,1% formic acid, 8 μm phosphoric acid). An EVOQ Elite^TM^ triple quadrupole mass spectrometer (Bruker, Billerica, MA) equipped with an ESI source was used for detection. Free CE and PL species were analyzed by multiple-reaction monitoring using CE: [MNH_4_]^+^ to *m*/*z* 369, 7 eV, 40 ms; FC: [M − H_2_O]^+^, 0 eV, 30 ms; PC: [MH]^+^ to *m*/*z* 184, 25 eV, 30 ms; LPC: [MH]^+^ to *m*/*z* 184, 22 eV, 40 ms; SM: [MH]^+^ to *m*/*z* 184, 20 eV, 40 ms; LPE: [MH]^+^ to [RCOO + 58]^+^, 17 eV, 50 ms; BMP: [MNH4]^+^ to [MG − H_2_O]^+^ of the respective MG, 23 eV, 70 ms; PI/LPI: [M − H]^−^ to fatty acids anions, 50 eV, 120 ms, and the resolution of Q1/Q3 were set to 0.7. Data acquisition was done by MS Work Station (Bruker). Data were normalized for recovery, extraction, and ionization efficacy by calculating analyte/internal standard ratios (arbitrary units (AU)) and expressed as AU/mg or AU/g liver.

### Isolation of total RNA and analysis of gene expression by quantitative real-time PCR (qPCR)

Liver tissues (∼50 mg) of *ad libitum*-fed (chow or VitA/HFD) hep-LAL-ko or LAL-del mice and respective WT littermates were homogenized in 1 ml of TRIzol using a ball mill. Primary hepatocytes and NPCs were lysed by addition of 500 μl of TRIzol and incubation at room temperature for 5 min. Phase separation was performed by addition of 100 μl of 1-bromo-3-chloropropane/ml of TRIzol and centrifugation at 12,000 × *g* at 4 °C for 15 min. Supernatant was transferred, and total RNA was precipitated by addition of 500 μl of isopropyl alcohol/ml TRIzol and centrifugation at 12,000 × *g* and 4 °C for 10 min. DNase-digested RNA was reverse-transcribed into cDNA using high capacity cDNA reverse transcription kit (Thermo Fisher Scientific). qPCR was performed as described previously (52). Primer sequences are listed in Table S2. Target gene expression was calculated by the ΔΔ*Ct* method. Expression of ribosomal housekeeping gene *36b4* was used for normalization.

### Analysis of protein expression levels by immunoblotting

Proteins of cell or tissue homogenates (10–30 μg of protein) were dissolved in SDS sample buffer, separated by 10% SDS-PAGE (10–12.5% Tris-glycine), and transferred onto a polyvinylidene difluoride membrane (Carl Roth GmbH, Karlsruhe, Germany). The membrane was blocked with 10% nonfat dry milk in TST (50 mm Tris-HCl, 0.15 m NaCl, 0.1% Tween 20 (pH 7.4)). The antibodies used are listed in Table S3. Protein expression was visualized using the ECL Plus Western blotting detection reagent (Thermo Fisher Scientific, Waltham, MA) and ChemiDoc Touch Imaging System (Bio-Rad).

### Measurement of in vitro RE hydrolase activity

The RE hydrolase activity assay was performed as described previously (9) with some modifications. In brief, 100 μl of cell lysates or liver homogenates (50 μg of protein) were incubated with 100 μl of substrate for 1 h at 37 °C. The substrate consisted of 300 μm RP and 300 μm PC. In some cases, the substrate additionally contained 100 μm PI and 150 μm BMP. For substrate preparation, lipids were dried under N_2_ and emulsified in 100 mm sodium acetate buffer (pH 4.5) by sonication. Then, 4% FA-free BSA was added and thoroughly mixed by vortexing. Substrate blank incubation was performed with solution A. After incubation, 1 ml of *n*-hexane and 200 μl of ethanol containing 1.14 μm retinyl acetate as internal standard was added. Then, the samples were vigorously vortexed, and phase separation was accelerated by centrifugation at 5,000 × *g* and 4 °C for 10 min. Upper organic phase (800 μl) was collected and dried in a speed vac. Samples were dissolved in 100 μl of methanol/toluene (1:1, v/v) and analyzed by HPLC-FD.

### Measurement of in vitro TG and CE hydrolase activity

The TG hydrolase activity assay was performed as described previously (9) with some modifications. In brief, 100 μl of cell lysates or liver homogenates (50 μg of protein) were incubated with 100 μl of substrate for 1 h at 37 °C. The TG substrate contained 300 μm triolein and 10 μCi/ml [^3^H]triolein and was emulsified with 45 μm PC and in some cases with 45 μm PC/PI (3:1, Mol/Mol), and 17 μm BMP. Lipids were dried under N_2_ and emulsified by sonication in 100 mm sodium acetate buffer (pH 4.5). Then 5% FA-free BSA was added and thoroughly mixed by vortexing. Reactions were terminated by addition of 3.25 ml of methanol/chloroform/*n*-heptane (10:9:7, v/v/v) and 1 ml of 0.1 m potassium carbonate (pH 10.5). Then, the samples were vigorously vortexed and centrifuged at 2,000 × *g* for 10 min. The radioactivity in 500 μl of the upper phase was determined by liquid scintillation counting. Substrate blank incubation was performed with solution A.

The CE hydrolase activity assay was performed in principle as described above for the TG hydrolase activity assay but using cholesteryl oleate as substrate. The CE substrate contained 300 μm cholesteryl oleate, 1 μCi/ml [^14^C]cholesteryl oleate and was emulsified with 90 μm PC/PI (3:1, Mol/Mol), and 34 μm BMP in 100 mm sodium acetate buffer (pH 4.5) including 5% FA-free BSA.

### Statistical analyses

Data are presented as mean + or ± S.D. Statistically significant differences were determined by Student's unpaired *t* test (two-tailed). Group differences were considered statistically significant for *p* < 0.05 (*), *p* < 0.01 (**), and *p* < 0.001 (***) or *p* < 0.05 (#), *p* < 0.01 (##), and *p* < 0.001 (###) between feeding regimens and time points.

## Author contributions

L. P., C. W., U. T., R. S., G. H., T. O. E., R. Z., and A. L. conceptualization; L. P., C. W., U. T., R. S., N. F., I .P., L.-M. P., and A. L. data curation; L. P., R. S., S. K., N. F., I. P., L.-M. P., B. I. W., and G. H. formal analysis; L. P. validation; L. P., C. W., R. S., S. K., N. F., I. P., G. S., L.-M. P., and B. I. W. investigation; L. P., C. W., R. S., N. F., I. P., P. V., and A. L. visualization; L. P., C. W., U. T., R. S., S. K., N. F., I. P., G. S., L.-M. P., T. O. E., and A. L. methodology; L. P. and A. L. writing-original draft; L. P., C. W., U. T., R. S., N. F., I. P., G. H., T. O. E., R. Z., and A. L. writing-review and editing; U. T., G. S., P. V., G. H., T. O. E., R. Z., and A. L. supervision; G. S., R. Z., and A. L. funding acquisition; G. H., T. O. E., R. Z., and A. L. resources; A. L. project administration.

## Supplementary Material

Supporting Information

## Footnotes

2 The abbreviations used are:

BMP: bis(monoacylglycero)phosphate
CAB: Chromotrop-Anilinblue trichrome
CE: cholesteryl ester
CESD: cholesteryl ester storage disease
CHOL: cholesterol
del: deleter
FA: fatty acid
FLP: flippase
H&E: hematoxylin and eosin
hep-LAL-ko: hepatocyte-specific LAL knockout
HFD: high fat diet
HPLC-FD: HPLC with fluorescence detection
LAL: lysosomal acid lipase
LIPA: lipase A = LAL
NEFA: non-esterified fatty acid
LAL: lysosomal acid lipase
LPC: lyso-PC
LPE: lyso-PE
LPI: lyso-PI
NPC: non-parenchymal cell
ORO: Oil Red O
PC: phosphatidylcholine
PE: phosphatidylethanolamine
PI: phosphatidylinositol
qPCR: quantitative real-time PCR
RE: retinyl ester
ROH: retinol
RP: retinyl palmitate
SM: sphingomyelin
TG: triglyceride
VitA: vitamin A.
