# Supplementary material for: Hepatocyte-specific deletion of lysosomal acid lipase leads to cholesteryl ester but not triglyceride or retinyl ester accumulation
Source: J Biol Chem. 2019 Apr 25;294(23):9118–33. doi: 10.1074/jbc.RA118.007201 (PMC6556574; doi:10.1074/jbc.RA118.007201)
Supplement: Supporting Information [file supp_294_23_9118__index.html]

Hepatocyte-specific deletion of lysosomal acid lipase leads to cholesteryl ester but not triglyceride or retinyl ester accumulation — Lysosomal acid lipase in neutral lipid metabolism — Hepatocyte-specific deletion of lysosomal acid lipase leads to cholesteryl ester but not triglyceride or retinyl ester accumulation — Lysosomal acid lipase in neutral lipid metabolism — Supporting Information 

# Hepatocyte-specific deletion of lysosomal acid lipase leads to cholesteryl ester but not triglyceride or retinyl ester accumulation

## Supporting Information

- Supporting Information (to be published online) - Supporting online material: Text, figures, and tables
