# Supplementary material for: Hepatocyte-specific deletion of lysosomal acid lipase leads to cholesteryl ester but not triglyceride or retinyl ester accumulation
Source: J Biol Chem. 2019 Apr 25;294(23):9118–33. doi: 10.1074/jbc.RA118.007201 (PMC6556574; doi:10.1074/jbc.RA118.007201)
Supplement: Supporting Information [file supp_RA118.007201_142643_2_supp_319829_pqh5ws.pdf]

Supporting Information

Hepatocyte-specific deletion of lysosomal acid lipase leads to cholesteryl ester but not triglyceride or retinyl ester accumulation

Laura Pajed[1], Carina Wagner[1], Ulrike Taschler[1], Renate Schreiber[1], Stephanie Kolleritsch[1], Nermeen Fawzy[1], Isabella Pototschnig[1], Gabriele Schoiswohl[1], Lisa-Maria Pusch[1], Beatrix I. Wieser[2], Paul Vesely[2], Gerald Hoefler[2,4], Thomas O. Eichmann[1,3], Robert Zimmermann[1,4], and Achim Lass[1,4*]

From the [1]Institute of Molecular Biosciences, NAWI Graz, University of Graz, Heinrichstraße 31/II, 8010 Graz, Austria; [2]Diagnostic & Research Center for Molecular BioMedicine, Institute of Pathology, Medical University of Graz, Graz, Austria; [3]Center for Explorative Lipidomics, BioTechMed-Graz, Graz, Austria; [4]BioTechMed-Graz, Graz, Austria

Running title**:** *Lysosomal acid lipase in neutral lipid metabolism*

[*]Corresponding author: Achim Lass, Institute of Molecular Biosciences, University of Graz, Heinrichstraße 31/II, 8010 Graz, Austria, Phone: +43 316 380 1900; Fax: +43 316 380 9016; E-mail: achim.lass@uni-graz.at

Experimental procedures

Quantification of plasma neutral lipids, glycerol, and β-hydroxybutyrate by colorimetric tests

Total CHOL (Cholesterol CHOD-PAP kit, Roche), TG (Triglyceride TM infinity kit, Thermo Scientific), NEFA (NEFA-HR(2) R1 and R2 Set, Wako Chemicals), glycerol (Free glycerol reagent, Sigma), and β-hydroxybutyrate (β-Hydroxybutyrate Colorimetric Assay Kit, Cayman Chemical) levels were analyzed by commercial enzymatic colorimetric kits according to manufacturer's instructions.

Isolation of total DNA and analysis of mitochondrial DNA content

Total DNA of liver tissues (20 mg) from *ad libitum* fed (chow or VitA/HFD) hep-LAL-ko mice and WT littermates was isolated using DNeasy Blood and Tissue Kit (Qiagen). Evaluation of relative copy number of mitochondrial DNA (mt-DNA) and nuclear DNA (n-DNA) was performed by qPCR according to (1). As marker gene of mt-DNA the *mitochondrial cytochrom c oxidase* (*Mt-Co1*) and of n-DNA the *NADH dehydrogenase flavoprotein 1* (*Ndufv1*) were amplified. Primer sequences are listed in Table S2. Expression levels were calculated using the ΔΔCt-method and relative mt-DNA copy numbers were analyzed by determining the mtDNA/n-DNA ratios.

Preparation of mitochondrial enriched fractions and fatty acid oxidation assay

Preparation of mitochondrial enriched fractions and determination of FA oxidation rates were performed as described (2). Briefly, liver (big lope) from hep-LAL-ko mice and WT littermates fed a standard chow diet or VitA/HFD were excised and washed in ice cold STE buffer (0.25 M sucrose, 10 mM Tris, 1 mM EDTA, 20 µg/ml leupeptin, 2 µg/ml antipain, and 1 µg/ml pepstatin). Tissues were homogenized by 4 strokes using a glass douncer and centrifuged for 10 min at 450 x *g* and 4°C. Infranatants, excluding floating fatty layer, were collected and aliquoted (=liver homogenate). For preparation of a mitochondrial enriched fraction, liver homogenates were centrifuged for 15 min at 3,000 x *g* and 4°C. Resulting pellets were washed twice in STE buffer and re-suspended in 100 µl STE buffer. Protein concentrations of liver homogenates and mitochondrial enriched fractions were determined by Bio-Rad protein assay (Bio-Rad) according to manufacturer's instructions using BSA as standard. For FA oxidation assay, mitochondria were incubated with the reaction mix (100 mM sucrose, 10 mM Tris, 5 mM KH_2PO_4, 0.2 mM EDTA, 0.3% BSA, 80 mM KCl, 1 mM $MgCl_2$, 2 mM L-carnitine, 0.1 mM malate, 0.05 mM CoA, 2 mM ATP, 1 mM DTT, 100 µM palmitic acid, and 0.1 µCi/reaction [1-^{14}C]-palmitic acid) for 30 min and 350 rpm at 37°. Then, the reaction mix was

transferred into a new tube containing 70% perchloric acid and a Whatman® paper soaked with 50 µl of 5 N NaOH placed into the lid. CO_2 was trapped for 60 min at RT. After removing the filter papers, tubes were centrifuged for 10 min at max. speed. The amount of ^{14}C in the CO_2 (filter papers) and acid soluble metabolites (ASM) (supernatants) fractions were determined by liquid scintillation counting.

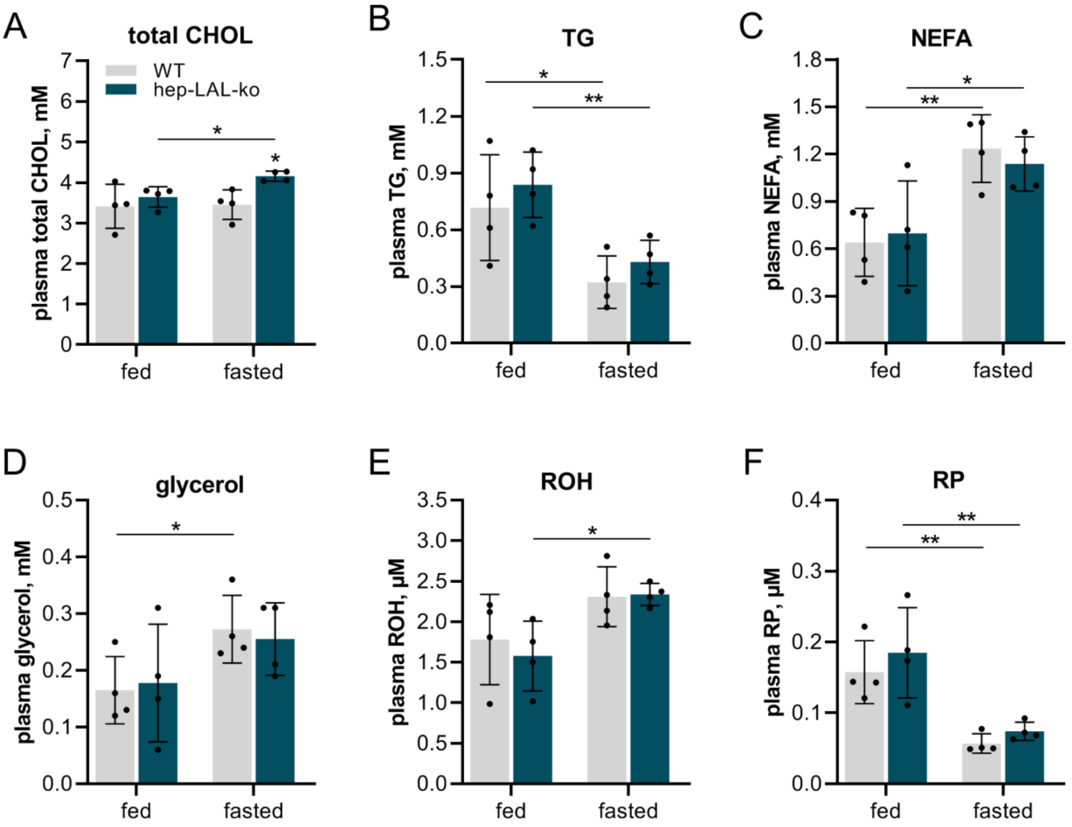

Figure S1. Unchanged plasma lipid and glycerol levels of mice lacking LAL specifically in hepatocytes. Blood was collected from *ad libitum* fed and overnight fasted, age- (5 months) and gender- matched (male) hepatocyte-specific LAL-deficient mice (hep-LAL-ko) and littermates (WT). (A) Total cholesterol (total CHOL), (B) triglyceride (TG), (C) non-esterified fatty acid (NEFA), and (D) glycerol plasma levels were determined by commercial kits. For the measurement of (E) retinol (ROH) and (F) retinyl palmitate (RP) plasma samples were *n*-hexane extracted and analyzed by HPLC-FD. Data are presented as mean ± S.D. for duplicate determinations (n=4 for all groups). Statistically significant differences were determined between genotypes and feeding status by Student´s unpaired *t*-test (two tailed; *.. $p < 0.05$, **.. $p < 0.01$).

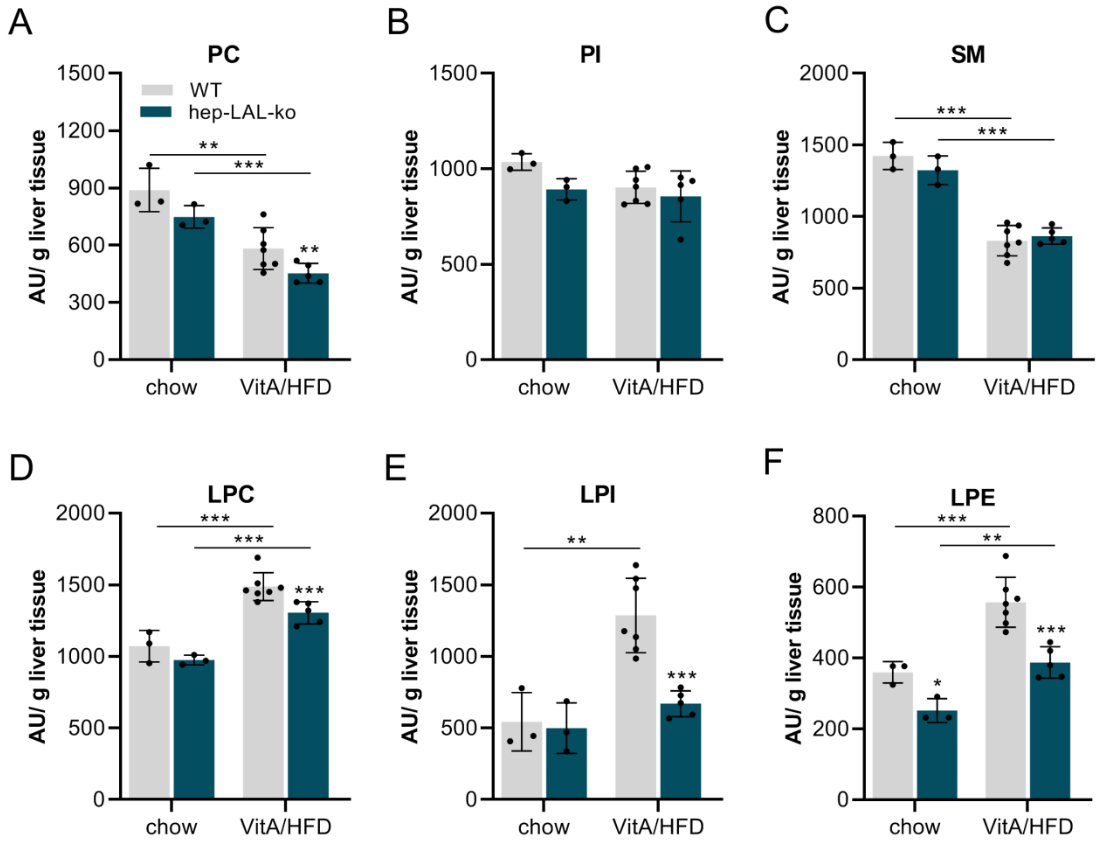

Figure S2: Vitamin A excess/HFD feeding decreases phospholipid levels in hepatocyte-specific LAL-deficient mice. Female hepatocyte-specific LAL deficient mice (hep-LAL-ko) and littermates (WT) 5 months of age were *ad libitum* fed a standard chow or vitamin A excess (100,000 IU VitA)/high fat diet (HFD) for 3 weeks. Lipids of liver homogenates were Folch-extracted and (A) phosphatidylcholine (PC), (B) phosphatidylinositol (PI), (C) sphingomyelin (SM), (D) lysophosphatidylcholine (LPC), (E) lysophosphatidylinositol, and (F) lysophosphatidylethanolamine (LPE) were analyzed by QQQ/MS. Date were normalized to g tissue and are presented as mean ± S.D. of duplicate determinations (n=3 of chow fed control mice; n=5 for hep-LAL-ko and n=7 for WT on VitA/HFD). Statistically significant differences were determined between genotypes and treatment by Student´s unpaired *t*-test (two tailed; *.. $p < 0.05$, **.. $p < 0.01$, ***.. $p < 0.001$).

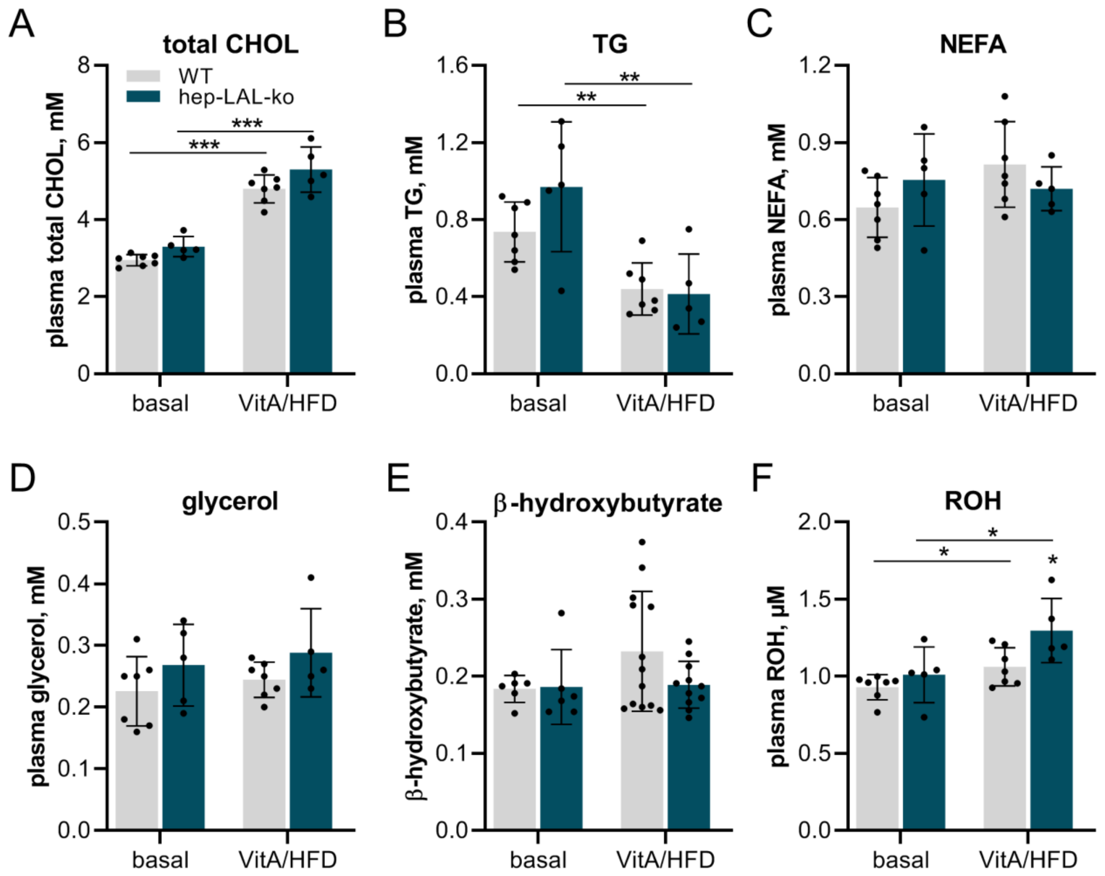

Figure S3. Vitamin A excess/HFD feeding leads to comparable changes in plasma lipid and glycerol levels of hepatocyte-specific LAL-deficient mice and WT littermates. Female hepatocyte-specific LAL deficient (hep-LAL-ko) mice and littermates (WT) 5 months of age were fed a vitamin A excess (100,000 IU VitA)/high fat diet (HFD) for 3 weeks. Blood was drawn from *ad libitum* fed mice before (=basal) and after 3 weeks of VitA/HFD feeding. (A) Total cholesterol (CHOL), (B) triglyceride (TG), (C) non-esterified fatty acid (NEFA), (D) glycerol, and (E) β-hydroxybutyrate levels were determined by commercial kits. For (F) retinol (ROH) measurement, plasma lipids were *n*-hexane extracted and analyzed by HPLC-FD. Data are presented as mean ± S.D. for duplicate determinations (n=5-11 for hep-LAL-ko and n=7-13 for WT mice). Statistically significant differences were determined between genotypes and treatment by Student´s unpaired *t*-test (two tailed; *.. $p < 0.05$, **.. $p < 0.01$, ***.. $p < 0.001$).

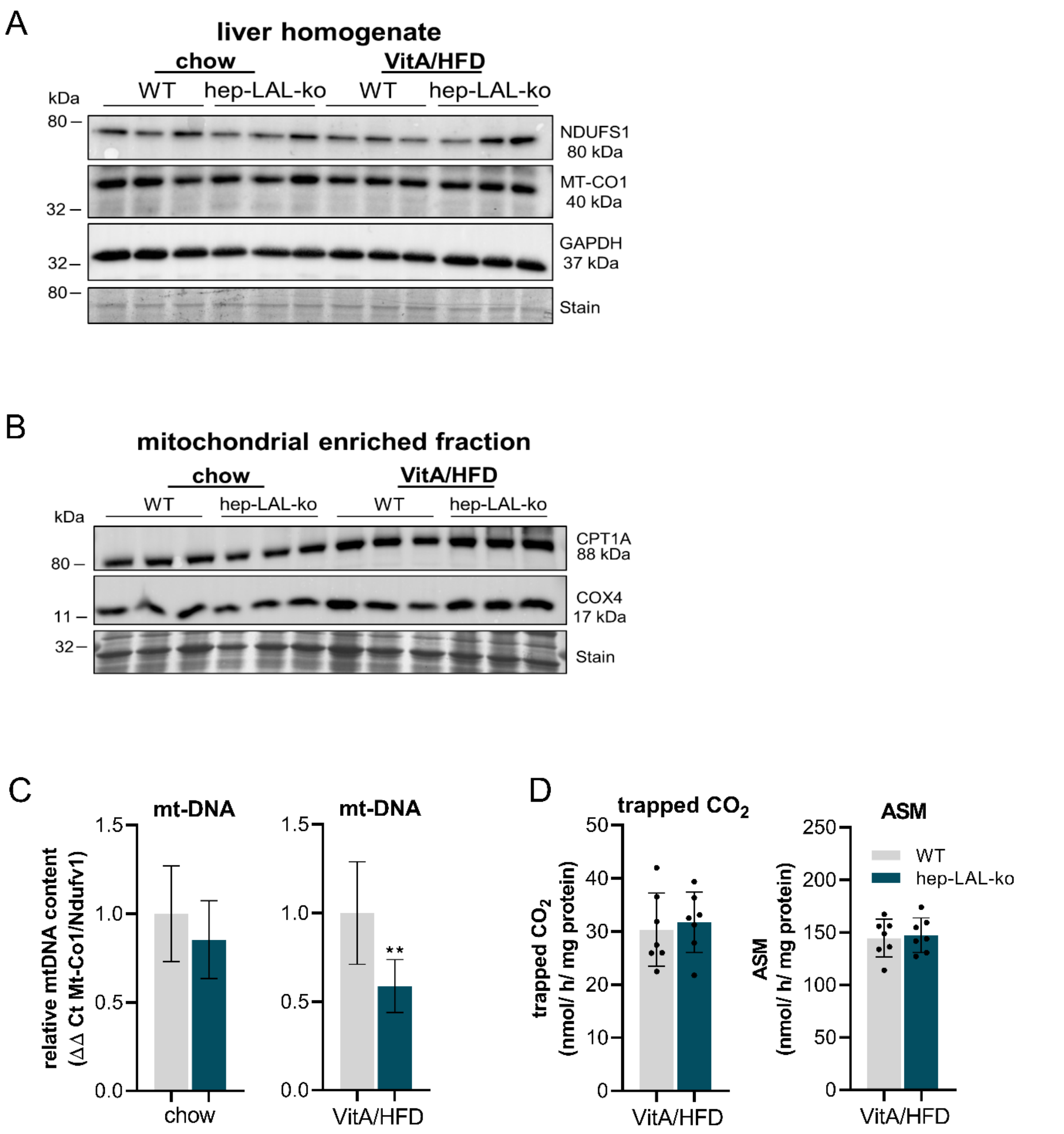

Figure S4: Mitochondrial protein content and fatty acid oxidation rate is unaltered in hepatocyte-specific LAL-deficient mice upon Vitamin A excess/HFD feeding. (A-C) Female hepatocyte-specific LAL deficient (hep-LAL-ko) mice and littermates (WT) 5 months of age were *ad libitum* fed a standard chow or vitamin A excess (100,000 IU VitA)/high fat diet (HFD) for 3 weeks. (A) Liver homogenates (450 × *g* supernatant) were prepared and subjected to Western blotting analyses using antibodies specific against mitochondrial marker protein NADH:ubiquinone oxidoreductase core subunit S1 (NDUFS1) and mitochondrially encoded cytochrome c oxidase I (MT-CO1). GAPDH and Coomassie blue stain were used as loading controls. (B) Liver was homogenized and mitochondrial enriched fractions (3,000 × *g* pellet) were prepared. Western blot analysis was performed using antibodies specific against carnitine O-palmitoyltransferase 1A (CPT1A) and loading control Cytochrome c oxidase 4 (COX4). (C) Total DNA of liver was isolated and qPCR was performed. Relative mt-DNA content was calculated from copy numbers of the mitochondrial encoded *Mt-Co1*/nuclear DNA-encoded *Ndufv1* gene ratios. (D) Male hep-LAL-ko and WT mice, 5 months of age, were fed a VitA/HFD for 3 weeks. Mice were fasted overnight, liver was freshly excised and mitochondrial enriched fractions were prepared. Fatty acid oxidation assay using ^{14}C-palmitic acid as substrate was performed. Trapped ^{14}C-CO_2 and ^{14}C-acid soluble metabolites (ASM) were determined by scintillation counting. Data are presented as mean ± S.D. for duplicate determinations (n=3-8 for

hep-LAL-ko and n=3-8 for WT on VitA/HFD). Statistically significant differences were determined between genotypes by Student´s unpaired *t*-test (two tailed; **.. $p < 0.01$).

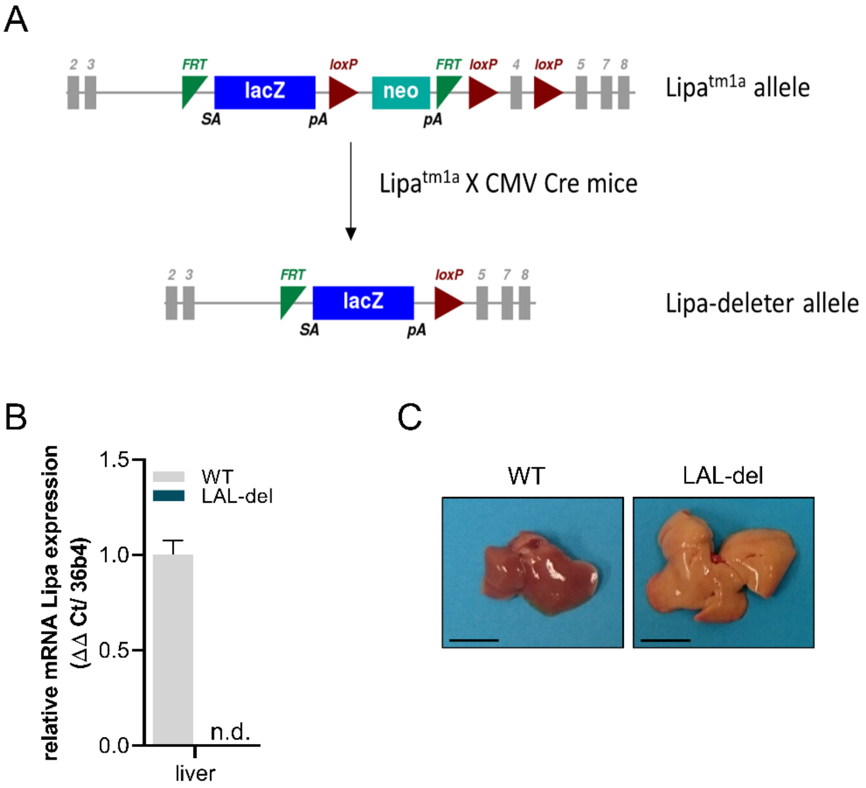

Figure S5. Breeding strategy for the generation of the LAL-deleter mouse strain (= mice globally lacking functional LAL) and validation of the LAL knockout. (A) Lipa^{tm1a} mice were crossed with mice expressing Cre recombinase under the control of the CMV promoter. Offspring positive for the Lipa deleter allele globally lack functional LAL (LAL-del). (B-C) Liver was excised. (B) mRNA was transcribed and expression of *Lipa* was determined by qPCR. (C) Macroscopic liver depiction. Scale bars represent 1 cm. Expression levels were calculated by the ΔΔCt-method using *36b4* as ribosomal housekeeping gene. *Lipa* expression was normalized to WT expression levels. Data are presented as mean + S.D. for duplicate determinations (n=4 for both LAL-del and WT mice). Abbreviation: n.d.= not-detectable

References

1. Quiros, P. M., Goyal, A., Jha, P., and Auwerx, J. (2017) Analysis of mtDNA/nDNA Ratio in Mice. *Curr. Protoc. Mouse Biol.* **7**, 47–54

2. Hirschey, M. D., Shimazu, T., Goetzman, E., Jing, E., Schwer, B., Lombard, D. B., Grueter, C. A., Harris, C., Biddinger, S., Ilkayeva, O. R., Stevens, R. D., Li, Y., Saha, A. K., Ruderman, N. B., Bain, J. R., Newgard, C. B., Farese Jr, R. V., Alt, F. W., Kahn, C. R., and Verdin, E. (2010) SIRT3 regulates mitochondrial fatty-acid oxidation by reversible enzyme deacetylation. *Nature*. **464**, 121–125

Table S1: Primer sequences used for determination of relative gene expression levels and mt-DNA and n-DNA contents.

Gene	Gene Name	NCBI Accession Number	Sequence of Primers
Abca1	ABC subfamily A member 1	NM_013454	F: 5'- CGTTTCCGGGAAGTGTCCTA -3'<br>R: 5'- GCTAGAGATGACAAGGAGGATGGA -3'
Abcg1	ABC subfamily G member 1	NM_009593.2	F: 5'- CCTTCCTCAGCATCATGCG -3'<br>R: 5'- CCGATCCCAATGTGCGA -3'
Acat2	Acetyl-Coenzyme A acetyltransferase 2 (=Soat2)	NM_146064	F: 5'- CCCGTGGTCATCGTCTCAG -3'<br>R: 5'- GGACAGGGCACCATTGAAGG -3'
Atgl	Adipose triglyceride lipase	NM_025802	F: 5'- GAGACCAAGTGGAACATC -3'<br>R: 5'- GTAGATGTGAGTGGCGTT -3'
Ctsd	Cathepsin D	NM_009983	F: 5'- GCTTCCGGTCTTTGACAACCT -3'<br>R: 5'- CACCAAGCATTAGTTCTCCTCC -3'
Cebpa	CCAAT/enhancer binding protein alpha	NM_007678	F: 5'- CAAGAACAGCAACGAGTACCG -3'<br>R: 5'- GTCACTGGTCAACTCCAGCAC -3'
Cd36	Cd36 molecule	NM_001159558	F: 5'- GAACCTATTGAAGGCTTACATCC- 3'<br>R: 5'- CCCAGTCACTTGTGTTTTGAAC- 3'
Cd68	Cd68 antigen	NM_001291058	F: 5'- ATCCCCACCTGTCTCTCTCA -3'<br>R: 5'- ACCGCCATGTAGTCCAGGTA -3'
Cpt1a	Carnitine palmitoyltransferase 1A	NM_013495	F: 5'- CACCAACGGGCTCATCTTCTA -3'<br>R: 5'- CAAAATGACCTAGCCTTCTATCGAA -3'
Cpt1b	Carnitine palmitoyltransferase 1B	NM_009948	F: 5'- CGAGGATTCTCTGGAACTGC -3'<br>R: 5'- GGTCGCTTCTTCAAGGTCTG -3'
Crebh	cAMP-responsive element-binding protein 3-like protein 3	NM_145365	F: 5'- TCGTGCCAGTGCGAGTGT -3'<br>R: 5'- AGCCACGCGGGATGC -3'
Dgat1	Diacylglycerol acyltransferase 1	NM_010046	F: 5'- GTGCACAAGTGGTGCATCAG -3'<br>R: 5'- CAGTGGGATCTGAGCCATCA -3'
Dgat2	Diacylglycerol acyltransferase 2	NM_026384	F: 5'- TTCCTGGCATAAGGCCCTATT -3'<br>R: 5'- AGTCTATGGTGTCTCGGTTGAC -3'
Elovl3	Elongation of very long chain fatty acids	NM_007703	F: 5'- ATGAATTTCTCACGCGGGTA -3'<br>R: 5'- GAGCTTACCCAGTACTCCTCCAAA -3'
Fabp4	Fatty acid binding protein 4	NM_024406	F: 5'- GAACCTGAAGCTTGTCTTCG -3'<br>R: 5'- ACCAGCTTGTCACCATCTCG -3'
Fasn	Fatty acid synthase	NM_007988	F: 5'- TCCTGGAACGAGAACACGATCT -3'<br>R: 5'- GAGACGTGTCACTCCTGGACTTG -3'
Fxr	Farnesoid X-activated receptor	NM_001163504	F: 5'- GCTTGATGTGCTACAAAAGCTG -3'<br>R: 5'- CGTGGTGATGGTTGAATGTCC -3'
F4/80	Adhesion G protein-coupled receptor E1	NM_010130	F: 5'- GGATGTACAGATGGGGGATG -3'<br>R: 5'- CATAAGCTGGGCAAGTGGTA -3'
Gk	Glycerol kinase	NM_008194	F: 5-' TGGTGGATGAGAGCTCAGTG -3'<br>R: 5-' TGAGCAGCACAAGTCGTACC -3'
G6Pase	Glucose-6-phosphatase	NM_008061	F: 5'- CCTCCTCAGCCTATGTCTGC -3'<br>R: 5'- AACATCGGAGTGACCTTTGG -3'
Hmg-CoA R	3-hydroxy-3-methylglutaryl-Coenzyme A reductase	NM_001360165	F: 5'- CCCTGAGTTTAGCCTTCCTTTTG -3'<br>R: 5'- GCTTTCTTTGAGGTCACGACGG -3'
Hmg-CoA S1	3-hydroxy-3-methylglutaryl-Coenzyme A synthase 1	NM_145942	F: 5'- CGGATCGTGAAGACATCAACTC -3'<br>R: 5'- CGCCCAATGCAATCATAGGAA -3'
Hmg-CoA S2	3-hydroxy-3-methylglutaryl-Coenzyme A synthase 2	NM_008256	F: 5'- AGAGAGCGATGCAGGAAACTT -3'<br>R: 5'- AAGGATGCCCACATCTTTTGG -3'
Hsl	Hormone sensitive lipase	NM_001039507	F: 5'- GCTGGGCTGTCAAGCACTGT -3'<br>R: 5'- GTAACTGGGTAGGCTGCCAT -3'
Lcad	Acyl-Coenzyme A dehydrogenase, long-chain	NM_007381	F: 5'- CCGGTTCTTTGAGGAAGTGAA -3'<br>R: 5'- AGTGTCGTCCTCCACCTTCTC -3'
Ldl-R	Low-density lipoprotein receptor	NM_001252658	F: 5'- TCAGTCCCAGGCAGCGTAT -3'<br>R: 5'- CTTGATCTTGGCGGGTGTT -3'
Lipa	Lysosomal acid lipase	NM_001111100	F: 5'- GGAACACTCGGTCCTGACAG -3'<br>R: 5'- CACATCAAAGCCAGCATCCG -3'
Lrat	Lecithin:retinol acyltransferase	NM_023624	F: 5'- ACAAGGAACGCACTCAGAAG -3'<br>R: 5'- GTCTAGGTGATTGACGAGGATG -3'
Lxr-a	Liver X receptor alpha	NM_001177730	F: 5'- CTCAATGCCTGATGTTTCTCCT -3'<br>R: 5'- TCCAACCCTATCCCTAAAGCAA -3'
Mcad	Acyl-Coenzyme A dehydrogenase, medium chain	NM_007382	F: 5'- GCAACTGCCCGCAAGTTT -3'<br>R: 5'- TACTCCCCGCTTTTGTCATATTC -3'
Mgl	Monoglyceride lipase	NM_011844	F: 5'- GATTTCACCTCTGGTCCTTG -3'<br>R: 5'- GTCAACCTCCGACTTGTTCC -3'

Mt-Co1	Mitochondrially encoded cytochrome c oxidase I	NC_005089.1	F: 5'- TGCTAGCCGCAGGCATTAC -3'<br>R: 5'- GGGTGCCCAAAGAATCAGAAC -3'
Ndufv1	NADH dehydrogenase flavoprotein 1	NM_001160038	F: 5'- CTTCCCCACTGGCCTCAAG -3'<br>R: 5'- CCAAAACCCAGTGATCCAGC -3'
Pgc1a	Peroxisome proliferative-activated receptor gamma coactivator 1 alpha	NM_008904.2	F: 5'- CCCTGCCATTGTTAAGACC -3'<br>R: 5'- TGCTGCTGTTCCTGTTTTC -3'
Pgc1b	Peroxisome proliferative-activated receptor gamma coactivator 1 beta	NM_133249.3	F: 5’- TCCTGTAAAAGCCCGGAGTAT -3'<br>R: 5'- GCTCTGGTAGGGGCAGTGA -3'
Ppara	Peroxisome proliferator-activated receptor alpha	NM_001113418	F: 5’- GTACCACTACGGAGTTCACGCAT -3’<br>R: 5’- CGCCGAAAGAAGCCCTTAC -3’
Pparg2	Peroxisome proliferator-activated receptor gamma 2	NM_001127330	F: 5'- CCAGAGCATGGTGCCTTCGCT -3'<br>R: 5'- CAGCAACCATTGGGTCAG -3'
Srebp1c	Sterol regulatory element-binding protein 1c	NM_001313979	F: 5'- GTTACTCGAGCCTGCCTTCAGG -3'<br>R: 5'- CAAGCTTTGGACCTGGGTGTG -3'
Srebp2	Sterol regulatory element-binding protein 2	NM_033218	F: 5‘- CCAAAGAAGGAGAGGCGG -3‘<br>R: 5‘- CGCCAGACTTGTGCATCTTG -3‘
Tgh	Carboxylesterase 1D (= triglyceride hydrolase)	NM_053200	F: 5’- GCCCTGGAGCTTCGTGAA -3’<br>R: 5’- CCTGCCCTCCAACAGCAT -3’
Vlcad	Acyl-Coenzyme A dehydrogenase, very long chain	NM_017366	F: 5’- CCGGTTCTTTGAGGAAGTGAA -3’<br>R: 5’- AGTGTCGTCCTCCACCTTCTC- 3’
Vldlr	Very low-density lipoprotein receptor	NM_001161420	F: 5’- GATGATGACGCAGACTGTTC -3’<br>R: 5’- CACTGGATCTCACTGGTAGG -3’

Table S2: Primer sequences for the genotyping of hep-LAL-ko mice, LAL-del, and respective WT littermates.

Primer	Sequences	Product size
LAL-flox	F: 5’- ATTGACAACAGCAGCTTGGG -3´<br>R: 5’- GCCAGGAATGGTAAGAATGCC -3´	WT allele: 360 bp;<br>floxed allele: 440 bp
Alb-Cre	F: 5’- GGGGTAGGAACCAATGAAA -3´<br>R: 5’- TAGCTGGCTGGTGGCAGATG -3	~1050 bp

Table S3: Antibodies used for immunoblotting.

Protein	Protein Name	Company
COX4	Cytochrome c oxidase subunit 4	Cell Signaling Technology (4844S)
CPT1A	Carnitine O-palmitoyltransferase 1A	Abcam (ab128568)
GAPDH	Glyceraldehyde 3-phosphate dehydrogenase	Cell Signaling Technology (2118S)
LAMP1	Lysosomal-associated membrane protein 1	Cell Signaling Technology (C54H11)
MT-CO1	Mitochondrially encoded cytochrome c oxidase I	Abcam (ab14705)
NDUFS1	NADH:ubiquinone oxidoreductase core subunit S1	Abcam (ab5521)
RAB7	Ras-related protein 7	Cell Signaling Technology (2094S)
Rabbit-HRP	HRP Goat Anti-Rabbit IgG Antibody	Vector Laborities (PI-1000)
Mouse-HRP	HRP Sheep Anti-Mouse IgG Antibody	GE healthcare UK (NA931V)
